# Shortwave-infrared meso-patterned imaging enables label-free mapping of tissue water and lipid content

**DOI:** 10.1038/s41467-020-19128-7

**Published:** 2020-10-23

**Authors:** Yanyu Zhao, Anahita Pilvar, Anup Tank, Hannah Peterson, John Jiang, Jon C. Aster, John Paul Dumas, Mark C. Pierce, Darren Roblyer

**Affiliations:** 1 Department of Biomedical Engineering, Boston University, 44 Cummington Mall, Boston, MA 02215 USA; 2 Beijing Advanced Innovation Center for Biomedical Engineering, School of Biological Science and Medical Engineering, Beihang University, Beijing, 100191 China; 3 Department of Pathology, Brigham and Women’s Hospital and Harvard Medical School, 75 Francis Street, Boston, MA 02115 USA; 4 Department of Biomedical Engineering, Rutgers, The State University of New Jersey, 599 Taylor Road, Piscataway, NJ 08854 USA

**Keywords:** Biomarkers, Preclinical research, Imaging and sensing, Near-infrared spectroscopy, Imaging techniques

## Abstract

Water and lipids are key participants in many biological processes, but there are few non-invasive methods that provide quantification of these components in vivo, and none that can isolate and quantify lipids in the blood. Here we develop a new imaging modality termed shortwave infrared meso-patterned imaging (SWIR-MPI) to provide label-free, non-contact, spatial mapping of water and lipid concentrations in tissue. The method utilizes patterned hyperspectral illumination to target chromophore absorption bands in the 900–1,300 nm wavelength range. We use SWIR-MPI to monitor clinically important physiological processes including edema, inflammation, and tumor lipid heterogeneity in preclinical models. We also show that SWIR-MPI can spatially map blood-lipids in humans, representing an example of non-invasive and contact-free measurements of in vivo blood lipids. Together, these results highlight the potential of SWIR-MPI to enable new capabilities in fundamental studies and clinical monitoring of major conditions including obesity, cancer, and cardiovascular disease.

## Abstract (web summary)

Quantifying lipid and water content in tissues non-invasively is difficult, and no method exists to quantify lipids in blood non-invasively. Here the authors develop an imaging approach called shortwave infrared meso-patterned imaging (SWIR-MPI) to detect and spatially map tissue water and lipids in preclinical models.

## Introduction

Water and lipids compose 60–80% of the human body and are inextricably linked to proper cellular function^1^. Alterations in the concentrations and spatial distribution of these components are hallmarks of many conditions including cardiovascular disease^2,3^, inflammation^4^, diabetes^5,6^, and several cancers^7^. Current imaging techniques such as magnetic resonance imaging (MRI) can generate tomographic volumes of fat and water contrast with T1 and T2 weighted scans, but are impractical for routine monitoring in the primary care setting. Techniques such as hyperspectral optical imaging are more accessible^8^, but cannot reliably quantify the concentrations of specific tissue chromophores owing to the confounding effects of optical absorption and scattering. Photoacoustic imaging (PA) largely avoids optical scattering effects by utilizing acoustic rather than optical detection. However, although PA has been used to probe in vivo lipid content, current works have been limited to lipid-rich tissues such as vascular plaques^9,10^. Few current imaging technologies can quantify tissue water content and none have been shown to noninvasively quantify lipids within circulating blood. Here we address this need through an optical imaging method that for the first time can quantify tissue water and lipid content through the skin and directly in superficial venous circulation.

Visible (VIS: 400–700 nm) and near-infrared (NIR: 700–900 nm) light has long been used to probe tissue composition in a non-invasive and label-free manner. For example, near-infrared spectroscopy (NIRS) is a common optical technique used to quantify oxy- and deoxyhemoglobin content for oncologic and functional brain imaging^11,12^. NIRS devices exploit the relatively weak optical attenuation of light within the so-called NIR optical window, which enables imaging depths of several centimeters for fiber-based point measurements, and several millimeters for wide-field imaging^13^. Unfortunately, in vivo quantification of water and lipid content has been limited by the dominating effects of hemoglobin absorption within the VIS and NIR spectral regions^14^. In contrast to blood, water and lipids each have distinct optical absorption characteristics at shortwave infrared (SWIR) wavelengths (900–2000 nm), suggesting that use of this spectral region may enhance the accessibility of these species.

To date, the role of water and lipids as stand-alone biomarkers has been somewhat underappreciated, with water absorption often considered a problem to be overcome in order to image more deeply with exogenous agents^15^. An additional barrier to implementation has been the limited availability of imaging detectors for the SWIR spectral region. Conventional silicon based cameras typically lack sensitivity past 1000 nm, and SWIR-sensitive InGaAs and germanium detectors have only recently become available in high resolution CCD or CMOS formats^16^. There has been recent interest in optical imaging in the SWIR spectral region^8,17^, but almost all prior work measured diffuse reflected light under simple planar illumination, which cannot reliably quantify tissue chromophores (e.g., water and lipids) owing to the confounding effects of optical scattering.

The ability to quantify molecular species in tissue with light is limited by the fact that optical absorption and scattering both contribute to overall imaging contrast. The inherent convolution of these physical effects severely constrains the ability of most wide-field optical imaging techniques to quantify light-absorbing chromophores such as hemoglobin, water, and lipids. Frequency-domain and time-domain diffuse optical techniques can isolate the effects of optical absorption and scattering using a combination of modulated illumination sources and model-based analysis, typically utilizing specific solutions to the diffusion approximation of the Boltzmann transport equation, or from Monte Carlo simulations^18,19^. After determining optical absorption at multiple wavelengths, the major light-absorbing components in the tissue (e.g., oxy- and deoxyhemoglobin, water, and lipids) can be quantified by solving a set of linear equations involving the known extinction spectra of each chromophore^20–24^. To date, no frequency-domain diffuse optical techniques have been developed to explore water and lipid contrast in the SWIR wavelength region.

Here, we develop an optical technique, SWIR Meso-Patterned Imaging, or SWIR-MPI, for quantitative, label-free optical imaging of water and lipids in tissue. SWIR-MPI uses a hyperspectral patterned illumination scheme that spans the water and lipid absorption bands from 900 to 1300 nm. A summary of SWIR-MPI imaging parameters including wavelength range, chromophore sensitivity, and spatial resolution are shown in comparison to other diffuse optical technologies in Supplementary Note 1^19,21–23^. In this work, we show how the reduced optical scattering of tissue in the SWIR region can be exploited to increase the imaging depth compared to the VIS or NIR regions, and demonstrate the ability to probe subcutaneous molecular and metabolic processes through intact skin. We demonstrate how SWIR-MPI can be used to monitor edema in an in vivo model of inflammation and quantify lipid heterogeneities in tumors. We also demonstrate that SWIR-MPI can noninvasively quantify and spatially map circulating lipids in superficial blood vessels in human subjects, enabling transient increases in lipids (i.e., lipemia) to be monitored following consumption of a high-fat meal. To the best of our knowledge, this is the first demonstration of label-free non-contact imaging of blood lipids through the human skin. We conclude with a discussion of basic science and clinical application areas in which SWIR-MPI may have a substantial impact.

## Results

### System design, imaging principles, and model-based data analysis

**Fig. 1:**
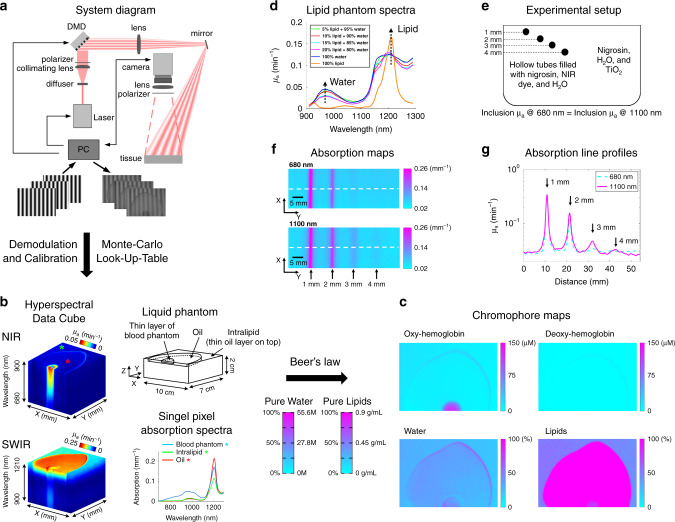
Hyperspectral SWIR-MPI system design and data flow. **a** A tunable NIR-SWIR laser illuminates a digital micromirror device (DMD) to generate meso-scale illumination patterns which are then projected onto the sample plane. Remitted light is collected by a germanium CMOS camera. **b** Raw reflectance images are demodulated to extract the tissue response at spatial frequencies of interest, in this case 0 mm^−1^ and 0.1 mm^−1^. A Monte Carlo based inversion model is used to extract optical properties (absorption and reduced scattering) on a pixel-by-pixel basis for each illumination wavelength to generate hyperspectral data cubes. Here, a liquid phantom (10 × 7 × 2 cm) was fabricated with an intralipid base partially coated with a layer of soybean oil and a thin layer of a blood (oxyhemoglobin)/TiO_2_ mixture. A thin layer of oil was formed on top of the intralipid base due to diffusion. Hyperspectral absorption (*µ*_a_ (mm^−1^)) data cubes are shown for the NIR and SWIR wavelengths bands. Hemoglobin absorption contrast is clearly apparent in the NIR region but only to a small degree in the SWIR region, whereas water and lipid contrast are both apparent in the SWIR band. **c** 2D maps of oxyhemoglobin, deoxyhemoglobin, water, and lipid concentrations are shown, highlighting the ability of SWIR-MPI to extract the spatial mosaic of chromophores present in the phantom. Following common practice in the diffuse optical spectroscopy field, water and lipid concentrations are reported as percentages (%)^20^. **d** Optical absorption (*µ*_a_) spectra of water-lipid phantoms over a range of concentrations; 100% water and lipid spectra are plotted for reference. **e** The measurement setup of a phantom imaging depth experiment (side view). **f** Optical absorption maps at 680 nm and 1100 nm. The absorption of the phantom was approximately equal at these two wavelengths, providing a means to compare the improved imaging depth at 1100 nm. **g**. Line profiles of the measured optical absorption corresponding to the dashed white lines in **f**, demonstrating improved contrast at 3 and 4 mm depths at 1100 nm. Source data are provided as a Source Data file.

Figure 1a shows the SWIR-MPI system diagram. The system utilizes a wavelength-tunable pulsed laser (effectively used as a continuous-wave (CW) light source) and a digital micromirror device (DMD) to generate planar and meso-scale patterned illumination at wavelengths from 680 to 1300 nm over a 10 cm × 7 cm sample plane. The wavelength-tunable laser provides high optical power (>800 mW) at each spectral band, which assisted in imaging water and lipids in the SWIR, which have as much as 10× the absorption of hemoglobin in the NIR region^25,26^. A VIS-NIR-SWIR-active germanium complementary metal oxide semiconductor (CMOS) camera is used to collect remitted light from the sample. The diffuse reflectance of the sample under illumination with different 1-D spatial frequencies is determined at each pixel in the image through demodulation and calibration steps (Supplementary Note 2)^19^. In this work, a combination of DC (0 mm^−1^) illumination and a meso-scale AC (0.1 mm^−1^) spatial frequency were used for all measurements. We have previously shown that this combination is effective for the extraction of optical properties (absorption and reduced scattering, denoted as *µ*_a_ and $\mu _s^\prime$, respectively) in a wide range of tissues^27,28^. These diffuse reflectance measurements are then used as input to an inverse model that calculates tissue optical properties on a pixel-by pixel basis. The inversion algorithm searches a pre-computed lookup table (LUT) generated by Monte Carlo simulations of light propagation in tissue^29^. The LUT maps diffuse reflectance values measured at the different spatial frequencies to unique combinations of *µ*_a_ and $\mu _s^\prime$. Beer’s law is then used to extract molar concentrations of dominant tissue chromophores (oxy- and deoxyhemoglobin, water, and lipids) based on the measured *µ*_a_ spectra and the known extinction spectrum of each chromophore^30^.

### Phantom validation studies

Figure 1b demonstrates the ability of SWIR-MPI to extract a complex spatial mosaic of chromophores in an optical phantom. In this example, a liquid phantom was fabricated from intralipid (an emulsified water/lipid solution), bovine blood, and soybean oil, with hyperspectral optical absorption data cubes generated for both NIR and SWIR wavelength regions. Areas containing hemoglobin show distinct absorption features at NIR wavelengths, whereas the areas with soybean oil show absorption features in the SWIR. Absorption spectra from single pixel locations highlight distinct features for blood, oil, and intralipid. The contribution of oxyhemoglobin absorption is clearly visible in the NIR region, whereas the 1210 nm absorption peak of lipid is clearly visible in the SWIR. The spectrum from the thin layer of blood also exhibits this lipid absorption feature, demonstrating a partial volume effect owing to the nature of diffusive photons, in which absorption from different tissue regions within the optical probing volume are effectively averaged. Figure 1c shows measured concentration maps of oxyhemoglobin, deoxyhemoglobin, water, and lipids in the optical phantom. Following common practice observed in the diffuse optical spectroscopy field, water and lipid concentrations are reported as percentages^20^. The water content is reported as the concentration of measured water divided by pure water concentration (55.6 M). The lipid concentration is reported as relative to an assumed “pure” lipid density of 0.9 g/ml^20^.

A series of homogenous phantoms with varying water and lipid content were measured with SWIR-MPI to validate the accuracy of chromophore content extraction. Absorption (*µ*_a_) and reduced scattering ($\mu _s^\prime$) spectra were measured from 910 nm to 1290 nm (Fig. 1d for *µ*_a_ spectra and Supplementary Fig. 3a for $\mu _s^\prime$ spectra), followed by extraction of water and lipid concentrations (Supplementary Table 3). The average error for water content estimation was −0.2 ± 2.5%, and the average error for lipid was 0.3 ± 1.6% over a wide physiologic range (5–20% for lipids, 80–95% for water). These results demonstrate that SWIR-MPI can extract water and lipid concentrations with high accuracy. We note that this accuracy was obtained in spatially homogenous samples, and it is expected that spatially complex tissues will induce a partial volume measurement effect, as demonstrated above in the phantom study (Fig. 1b, c). In addition, a drift test was conducted over 5 h to demonstrate measurement robustness (Supplementary Note 3).

### SWIR-MPI imaging depth

Non-invasive in vivo applications typically require imaging through intact skin. Within the 900–1300 nm region, a SWIR imaging window centered ~1100 nm was identified which enables deep tissue imaging while still capturing the relevant spectral features of water and lipids (Fig. 1e–g and Supplementary Fig. 3b–d). We quantified the improvement in optical penetration depth within this window compared to the NIR by using Monte Carlo simulations (Supplementary Note 4) and phantom experiments (Supplementary Table 5). SWIR illumination at 1100 nm provided a 55% increase in contrast at a depth of 3 mm in phantoms compared to NIR illumination at 680 nm, and a 20% increase in contrast at 4 mm depth. The increased penetration depth compared to VIS or NIR wavelengths is largely due to decreased optical scattering in the SWIR region^8,31–35^, and provides a strong advantage in imaging subcutaneous tissue water and lipids, as well as lipids within large superficial blood vessels, which may lie 1–2 millimeters below the skin surface^36,37^.

### In vivo water content monitoring

The accumulation of tissue water in the extravascular interstitial space (i.e., edema) is associated with the normal tissue healing process, inflammation, and a wide range of pathologies^3^. Despite its physiologic importance, there is currently a lack of standardized methods to assess the presence, extent, and time course of edema. We demonstrate here how SWIR-MPI can help address this need.

**Fig. 2:**
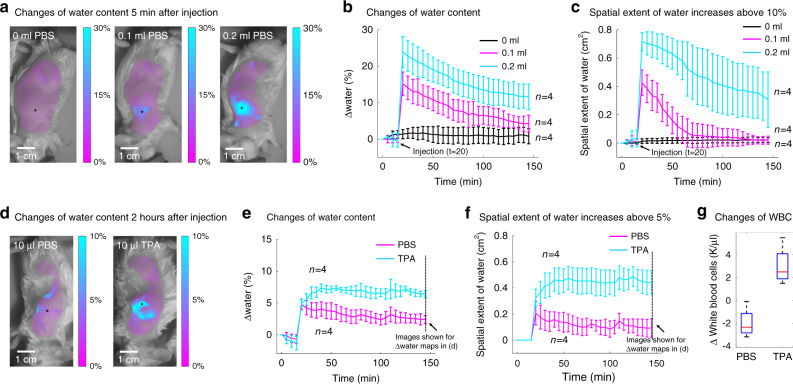
Non-invasive monitoring of tissue water content in an in vivo model of edema and acute inflammation. **a** Bolus injections of PBS were given subcutaneously to BALB/c mice at three dosages (*n* = 4 mice per group). Representative mice are shown 5 min after injection. The injection site is indicated by an asterisk. **b** Time series of mean water changes for each group (error bars indicate standard deviations). For the 0.1 ml and 0.2 ml groups, significant water changes were observed after the injection (*p* = 0.001 and *p* = 0.004, respectively). **c** Time series of mean spatial extent of water content increases. For the 0.1 ml and 0.2 ml groups, significant changes in spatial extent were observed after the injection (*p* = 0.003 and *p* < 1e-3, respectively). **d** Bolus injections of 10 µl PBS (control) or 10 µl TPA (pro-inflammatory) were given subcutaneously in two groups of mice (*n* = 4 mice per group). Changes in water content are shown 2 h after injection. A sustained increase of water content was observed in the TPA group. **e** Time series of changes in tissue water. For the 10 µl PBS group, the water content gradually decreased after the initial injection. For the 10 µl TPA group, water content increased for up to 30 min after injection, consistent with the presence of edema caused by inflammation, which was confirmed by histologic visualization of resected tissue. At 145 min, the water content of the TPA group remained elevated and significantly different from the control (PBS) group (*p* < 1e-4). **f** Time series of mean spatial extent of water content increases. At 145 min, the TPA group remained elevated and significantly different from the control (PBS) group (*p* < 1e-3). **g** The TPA group showed a significant increase in white blood cell count (WBCs) compared with the control group (*p* = 0.004) at 5 h after injection, indicating inflammation. A small decrease in WBCs was observed in the PBS group, consistent with previous measurements in mice under anesthesia^74^. Source data are provided as a Source Data file.

We first tested the ability of SWIR-MPI to track in vivo subcutaneous tissue water content in mice. Edema was simulated using subcutaneous injections of phosphate-buffered saline (PBS; 0 ml, 0.1 ml, or 0.2 ml) in the flank of BALB/c mice. SWIR-MPI longitudinally tracked water content for 2 h post injection. Figure 2a shows the spatial distribution and magnitude of water content changes immediately after injection, demonstrating a clear dose-dependent effect. Water changes are shown as a percentage of pure water. Figure 2b shows a time series of water content measurements from the injection site for all mice (*n* = 4 per group). The 0 ml group exhibits stable tissue water content over the 2-hour period, whereas the 0.1 ml and 0.2 ml groups show rapid increases immediately after injection, followed by gradual decreases toward baseline. For these mice, the average absolute baseline tissue water concentration before the injections was 56.9% ± 6.7%. The magnitude of the immediate post-injection water concentration for the 0.1 ml injection group was 69.8% ± 4.7%, and for the 0.2 ml injection group was 77.0% ± 9.7%. In addition, the spatial extent (in cm^2^) of increased water content caused by the injection was quantified over the 2-hour period. This spatial extent was defined as the area near the injection site that exhibited a change in water content (Δ water (%)) above specific threshold values. Figure 2c shows the temporal dependence of the spatial extent for all three groups of mice. A 1 cm diameter area around the injection site was used as a region of interest (ROI) to quantify the spatial extent of water content increases over 10% (as a percentage of pure water). Similar to the water content changes in Fig. 2b, the 0 ml group exhibits negligible spatial extent in terms of water content increase, whereas the 0.1 ml group and the 0.2 ml group show rapid increase of spatial extent immediately after injection, followed by gradual decreases toward baseline. For these mice, the average baseline spatial extent of water increases before the injections was 0.02 ± 0.04 cm^2^. The immediate post-injection spatial extent of water increases was 0.4 ± 0.1 cm^2^ for the 0.1 ml group, and 0.7 ± 0.1 cm^2^ for the 0.2 ml group. The observation that injections of nearly pure water do not lead to a linear increase in the measured water content (or 100% post injection tissue water concentrations) reflects the diffusion of PBS away from the injection site, and that the optical measurements incur a partial volume effect, in which the injected solution, the cutaneous tissue, and a portion of the subcutaneous tissue are integrated in the optical measurement causing a spatial averaging effect.

### Longitudinal tracking of inflammation-induced edema

Next, we tested the ability of SWIR-MPI to monitor transient peripheral edema induced by acute inflammation after the injection of 12-O-Tetradecanoylphorbol 13-acetate (TPA). Figure 2d shows changes in water content 2 h after the injection of TPA or control (PBS). Only a slight increase of water content was observed in control mice, whereas a sustained increase was observed in mice injected with TPA after 2 h. In the control group, the water content gradually decreased toward baseline over the 2-hour period (Fig. 2e, f). In contrast, water content continued to increase after the TPA injection for ~30 min, and then stayed relatively stable until the end of monitoring, indicating the induction of edema. The presence of inflammation was confirmed with white blood cell (WBC) counts from blood collected from the tail vein before and 5 h after PBS or TPA injections. WBCs increased substantially in the TPA group compared with the control group (*p* = 0.004) (Fig. 2g and Supplementary Table 6). Tissue samples were collected from the injection site and evaluated for immune cell infiltration by a board-certified pathologist who was blinded to the source of each tissue section (Supplementary Table 7). Tissues from the TPA group showed early signs of acute inflammation, whereas no acute inflammation was observed in the control group.

### Ex vivo mapping of tumor lipid content

Phenotypic and functional heterogeneity are known consequences of genetic and micro-environmental variations in space and time within a tumor^38^. It was recently demonstrated that lipid heterogeneity is an important biomarker for cancer diagnosis^39^, suggesting that this may be an underexplored prognostic factor in cancer. We show that SWIR-MPI can map lipid content across the tumor surface in resected specimens, providing a new means to estimate tumor lipid heterogeneity.

**Fig. 3:**
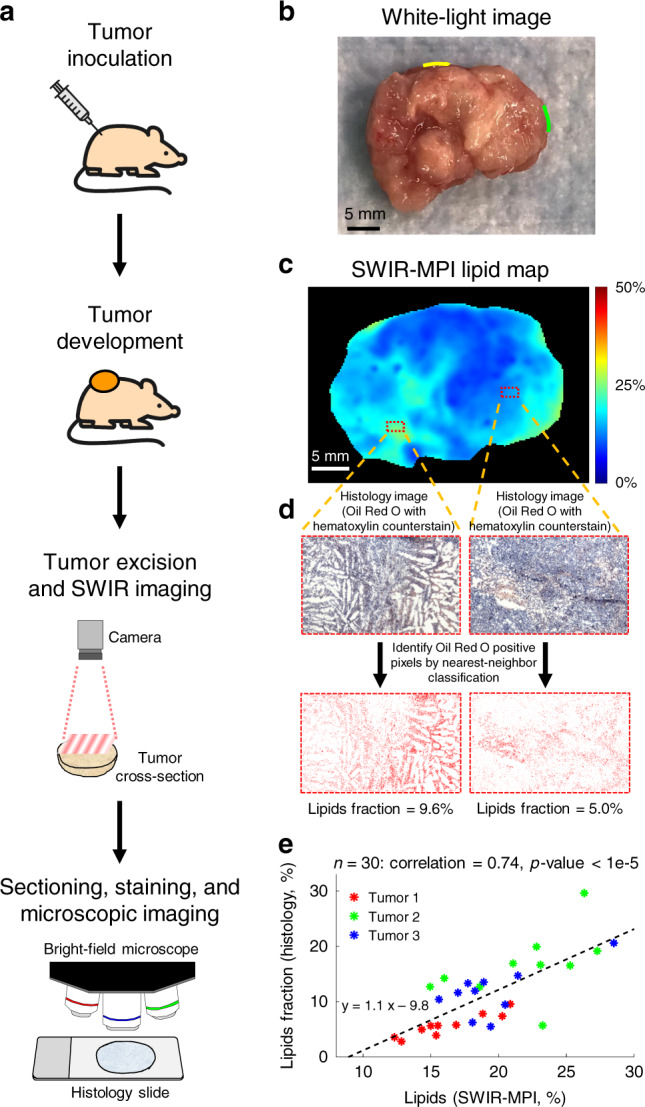
Ex vivo mapping of tumor lipid heterogeneity. **a** SCID mice were inoculated with PC3/2G7 prostate tumor cells. After tumors reached 3,000 mm^3^ they were excised, bisected, and the cross-section was imaged with SWIR-MPI. Tumors were then sectioned, stained with Oil Red O, and imaged under bright-field microscopy for lipid fraction analysis. **b**. White-light image of a representative tumor cross-section. Green and yellow marks were made with permanent inks to aid in co-registration with histology (digitally added to the image here). **c** Lipid content map generated with SWIR-MPI. The area within the red box was spatially co-registered to the Oil Red O histology slide (counterstained by hematoxylin) for comparison. **d** A binarized lipid map was generated using nearest-neighbor classification from the histology data in order to calculate lipid area fraction. The lipid fraction was calculated as the number of Oil Red O positive pixels over the total number of pixels within the ROI (2.2 mm × 1.2 mm). **e** Correlation plot of lipid content obtained by SWIR-MPI and Oil Red O histology. Each data point in the plot corresponds to a sub-region on the histology slide. The black dashed line indicates the best fit line and data from different tumors are indicated by color. The Pearson correlation coefficient is indicated. Source data are provided as a Source Data file. The pictures of the mouse and needle in **a** were generated using Google AutoDraw.

Figure 3 demonstrates ex vivo mapping of lipid content in a subcutaneous tumor model using SWIR-MPI. Visually occult spatial heterogeneities in lipid distribution are revealed over the tumor cross section (Fig. 3b, c). Imaging results were compared with Oil Red O staining followed by quantitative analysis (Fig. 3c, d, and Supplementary Fig. 5). The correlation between histology and SWIR-MPI measurements taken across 30 regions of interest (ROIs) from three tumors is shown in Fig. 3e. The choice of ROI was blinded from SWIR-MPI lipid maps. A Pearson correlation coefficient of 0.74 (*p* < 1e-5) was calculated from the data. Although SWIR-MPI is a relatively superficial imaging technique, typically probing the first several millimeters of tissue depth, it integrates lipid content lying more deeply than the histologic staining, which likely reduced the correlation strength. Despite this limitation, a relatively strong correlation between SWIR-MPI and the histopathologic gold standard is clearly present.

### In vivo identification of BAT

**Fig. 4:**
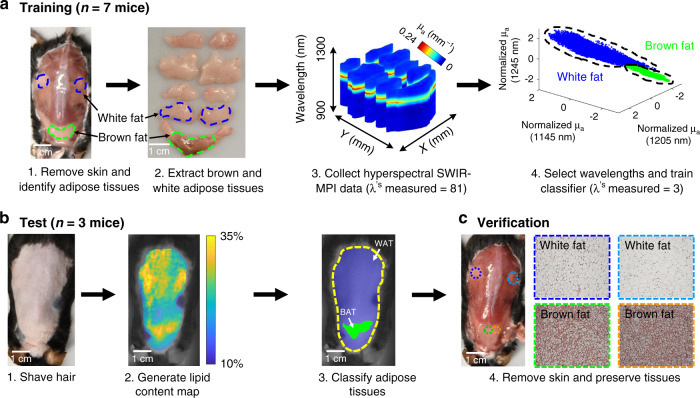
In vivo classification of brown adipose tissue. SWIR-MPI was integrated with a machine learning framework for identification of in vivo brown fat through intact skin. **a** In the algorithm training phase, mouse skin was removed from the dorsal region and brown and white adipose tissues were extracted (*n* = 7 mice). Hyperspectral (900–1300 nm) SWIR-MPI data were collected on ex vivo tissues. A feature selection algorithm identified three wavelengths with which to train a support vector machine (SVM) classifier. **b** In the test phase, SWIR-MPI data were collected at the three selected wavelengths from live anesthetized mice with shaved hair and intact skin (*n* = 3 mice). Regions of high-fat content were classified as either brown or white fat based on the trained SVM classifier. In each case, the intrascapular regions were classified correctly as brown fat. **c** For verification, mice were killed and dorsal skin was removed. Adipose tissue samples were collected, stained with H&E, and imaged with bright-field microscopy. The results matched with the SVM classifier predictions, which was confirmed by histologic visualization of resected tissues.

Brown and white adipose tissue (BAT and WAT, respectively) are the two major types of adipose tissue in mammals. BAT is well-known for its role in thermoregulation and anti-obesity^40,41^, while WAT is primarily responsible for energy storage and thermal insulation^42^. Here we show that SWIR-MPI integrated with a machine learning framework can noninvasively map and differentiate BAT from other tissues types in vivo without exogenous contrast. First, absorption maps of the posterior surface of seven C57BL/6 mice were measured at 81 wavelengths equally spaced between 680 and 1300 nm. Samples of brown and white adipose tissue were dissected and imaged with the same wavelengths (Fig. 4a, steps 2–3). A feature selection algorithm was used to rank the importance of the wavelengths and identify a subset of wavelengths that exhibited the potential to discriminate between BAT and other tissue types^43^. A support vector machine (SVM) classifier was then trained with the top three ranking wavelengths (1145 nm, 1205 nm, and 1245 nm) and used to classify brown and white fat data in the training set (Fig. 4a, step 4).

The SVM classifier was then applied to a test set consisting of three additional mice. SWIR-MPI data collected on these mice were acquired in vivo through intact skin with the three identified wavelengths, and corresponding lipid content maps were generated (Fig. 4b, steps 1–2). Regions of high-fat content were classified as either brown or white fat based on the trained SVM classifier (Fig. 4b, step 3). The flowchart for pixel-wise brown and white fat classification is shown in Supplementary Fig. 6. Pixels identified as brown fat were rendered in green on top of the white-light mouse image, whereas the white fat pixels were rendered in blue. The identified brown fat region agrees well with the known anatomical distribution of the interscapular BAT in mice^42^. To further validate the classification results, after imaging, the mouse skin was removed and tissue samples were collected from predicted BAT and WAT areas for Hematoxylin and Eosin (H&E) staining. The classification was confirmed by a board-certified pathologist via histologic visualization of the resected tissues (Fig. 4c, step 4). All other test set data are shown in Supplementary Fig. 7. In each case, the intrascapular regions were classified correctly as brown fat and confirmed as such by the pathologist.

### Non-invasive blood lipids monitoring in humans

Dyslipidemias (i.e., abnormal blood lipid levels) commonly manifest in patients suffering from diabetes, obesity, hypertension, and a variety of genetic diseases^44,45^. Blood lipids are commonly measured using invasive blood draws followed by centralized lab-based testing. We demonstrate here that the ratio of SWIR-MPI measured absorption at 1210 nm and 970 nm can noninvasively probe blood lipids in superficial venous vessels through intact skin in human subjects. Hereafter, we refer to the absorption ratio of 1210 nm over 970 nm as the SWIR-MPI index.

**Fig. 5:**
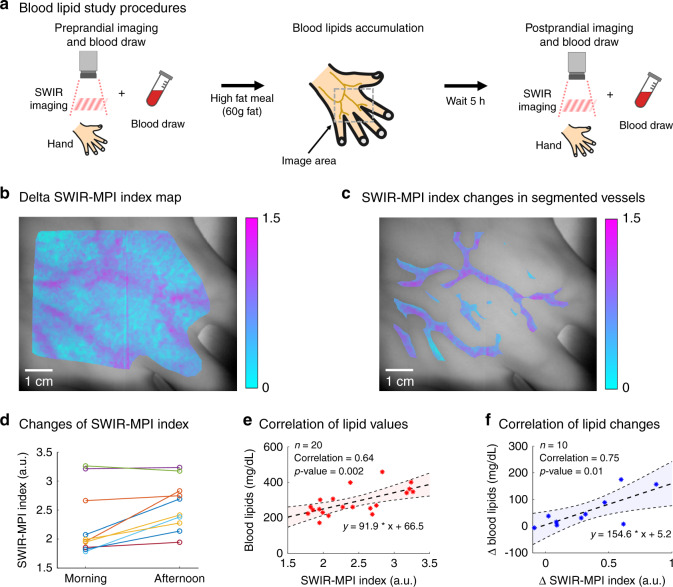
Pre- and post-prandial blood lipid measurement in human subjects. **a** Outline of study protocol. Normal subjects were measured with SWIR-MPI in the morning after fasting for 10 h overnight. A blood draw was taken immediately after imaging. Subjects then consumed a high-fat meal (containing 60 grams of fat). A second SWIR-MPI measurement and blood draw were conducted 5 hours later. Blood lipids (triglycerides and cholesterol) were determined from blood draws and used as the gold standard. **b** A representative map of change in SWIR-MPI index on the back of a subject’s hand. Major blood vessels (subcutaneous veins) are clearly visible. **c** Change in SWIR-MPI index in automatically segmented blood vessels. **d** Changes in SWIR-MPI index for ten individual subjects. Nine out of the ten subjects exhibited an increase in SWIR-MPI index over the 5-hour period after eating the high-fat meal, consistent with blood draw results (supplementary Table 8). **e** Correlation between SWIR-MPI index and absolute lipid values obtained from blood draws (Pearson correlation coefficient: 0.64). The shaded area indicates 95% confidence bounds for the data fit. **f** Correlation between changes in SWIR-MPI index and changes in absolute blood lipid values over the 5-hour period (Pearson correlation coefficient: 0.75). The shaded area indicates 95% confidence bounds for the data fit. Source data are provided as a Source Data file. The picture of the hand in **a** was generated using Google AutoDraw.

Figure 5a shows the study procedure. SWIR-MPI measurements of the dorsal surface of each healthy subject’s hand were collected after fasting for 10 h overnight and then again 5 hours after consuming a high-fat meal. It has been previously established that increases in blood lipids (i.e., lipemia) occur 3.5–7 h after a high-fat meal^46^. Blood was collected immediately following each imaging session and used to obtain lipid profiles for all subjects. The test subjects continued with their daily routine between the measurements and could drink water, but were advised not to eat other meals. In addition, no exercise was reported from the volunteers between measurements.

In order to visualize post-prandial blood lipid changes, superficial veins were automatically segmented by Hessian-based filtering on total hemoglobin concentration maps. The SWIR-MPI index difference maps (generated by spatially registering and subtracting the preprandial SWIR-MPI index map from the post-prandial index map) were then superimposed on the segmented vessels. Example maps showing the pre- to post-prandial SWIR-MPI index changes are shown for a representative subject (Fig. 5b, c), which demonstrates increases in lipids within the superficial large veins in this subject. In order to estimate blood lipids for each subject, SWIR-MPI index values were calculated from the segmented vessel pixels corresponding to the highest 10% hemoglobin concentrations. This was done to better isolate the most superficial vein segments in which overlying tissue had a minimal effect on the optical measurement of vessels under the skin. An increase in post-prandial SWIR-MPI index levels was observed in the same nine out of ten subjects with SWIR-MPI (Fig. 5d) and invasive blood draws (Supplementary Table 8). Absolute total blood lipids from blood draws and SWIR-MPI index were strongly correlated (Pearson correlation coefficient = 0.64, Fig. 5e), with an average percentage error of 3.5 ± 19.7% (eight of ten subjects had errors in the following range: (−28.8, 22.5%), two subjects had ~40% error). The average magnitude of percent error was 15.0%. In addition, when only the intra-subject changes of lipids before and after the meal were considered, invasive blood draws and SWIR-MPI again had a strong correlation (Pearson correlation coefficient = 0.75, Fig. 5f).

## Discussion

We have developed and validated SWIR-MPI as a new method to noninvasively image tissue water and lipid content in a label-free manner. This was enabled through the use of shortwave infrared light at wavelengths from 900 to 1300 nm where water and lipids are the dominant tissue chromophores. Meso-scale patterned illumination in combination with a light-transport model allows for separation of optical absorption and scattering effects, enabling quantification of tissue water and lipids. We demonstrated how SWIR-MPI could be used to longitudinally monitor in vivo water content through intact skin during inflammation-induced peripheral edema, and showed how SWIR-MPI can quantify occult lipid heterogeneities in resected tumors, and then how it can classify BAT through intact mouse skin in vivo. Finally, we demonstrated that a SWIR-MPI-derived index can noninvasively probe and spatially map blood lipid content in human subjects in vivo, with transient changes confirmed by invasive blood draws.

SWIR-MPI enjoys a number of advantages over methods currently used for quantifying the water and lipid content of tissue. Compared with popular analytical techniques, SWIR-MPI can be used in vivo through intact skin, a feature not shared by histology, multi-frequency impedance, or mass spectroscopy^47–49^. Compared with clinical imaging techniques such as MRI, SWIR-MPI is substantially less-resource intensive and can directly quantify concentrations of lipids and water. Competing optical methods, including fluorescence spectroscopy and imaging, rely on exogenous agents to label lipids and do not simultaneously quantify water^50^. In comparison to prior related techniques, such as NIR diffuse optical spectroscopic imaging (DOSI) and diffuse optical tomography (DOT), SWIR-MPI is non-contact and inherently wide-field^51^. These advantages enabled segmentation of superficial blood vessels for measurement of blood lipids in this work, which would be much more challenging with a point-probe based modality like DOSI. In the future, the combination of advantages enjoyed by SWIR-MPI could enable applications including tumor margin detection and determination of margins for surgical removal/debridement of infected or necrotic tissue. Recently, there have been a number of photoacoustic (PA) imaging studies of lipids^9,10,52–54^, and to a lesser degree, water^55^. Although PA approaches have several advantages, such as depth sectioning and/or tomographic reconstructions, the current state of the art for PA lipid imaging has been largely limited to tissues with highly focal, lipid-rich regions (e.g. lipid-rich plaques in the major coronary vessels). In addition, quantitative measurement of tissue lipid content remains an unmet challenge for PA. This is due at least in part to the fact that PA requires strong target absorption contrast, and that lipids and water have similar absorption strength at relevant wavelength bands^9,10,52^. This is in contrast to hemoglobin PA imaging in the VIS-NIR wavelength band, in which hemoglobin provides an extraordinarily high absorption contrast compared to background. In addition, to the best of our knowledge, PA imaging has not been reported for blood lipid measurements.

This work has several important implications for basic science and translational applications. For example, the presence and duration of peripheral edema is directly linked to clinical outcome for patients suffering from heart failure, burn wounds, soft tissue trauma, diabetes, as well as those recovering from surgery and radiation therapy^4,6,56,57^. Diagnosis of edema often relies on simple clinical observations, including the presence of skin redness, an increase in tissue volume, and pain to the touch^3^. We have shown that SWIR-MPI can quantitatively track both the induction and resolution of edema through measurement of tissue water content, suggesting a new means to quantify degrees of peripheral edema. In addition, SWIR-MPI may also be a useful tool for the study of cancer and obesity. We showed that SWIR-MPI can spatially map the distribution of lipids in ex vivo tumor samples, which points to applications in surgical margin detection and ex vivo diagnosis. We also showed that SWIR-MPI can identify subcutaneous brown fat (BAT). BAT has recently emerged as an imaging and therapeutic target with BAT thermogenesis currently viewed as an appealing approach to prevent or treat obesity and associated diseases including diabetes^5,58^. Current methods for measuring BAT in vivo, including MRI and PET/CT, are resource intensive and may require exogenous agents or ionizing radiation^59^. SWIR-MPI provides an attractive alternative for in vivo studies of BAT in small animals such as mice^60^.

Perhaps most importantly, we demonstrated that SWIR-MPI can noninvasively probe blood lipids through intact skin in human subjects. This ability has the potential to profoundly affect the diagnosis and longitudinal monitoring of dyslipidemia (i.e., abnormal blood lipid levels), which occurs in patients suffering from diabetes, obesity, hypertension, and a variety of genetic diseases^44,45^. Blood lipid levels, composed of cholesterol, triglycerides, and lipoproteins, are used to monitor cardiovascular disease progression, providing feedback for diet and exercise regimens as well as pharmacologic interventions^61^. Both overtreatment and undertreatment for hyperlipidemia are frequent in clinical practice, due in part to a lack of point-of-care methods for blood lipid monitoring^62^. The alternative of invasive blood draws with label-free SWIR-MPI could have a substantial impact on clinical practice and the 423 million patients worldwide suffering from cardiovascular disease and related ailments^63^.

SWIR-MPI has several limitations of note. For example, despite the fact that SWIR-MPI has the potential to image more deeply than systems that utilize VIS-NIR wavelengths, imaging depth remains limited to ~4 mm in biological tissues. This currently precludes the use of SWIR-MPI for non-invasive imaging of deeper internal organs, although development of minimally-invasive endoscopic SWIR-MPI could overcome this limitation. In addition, owing to the nature of light diffusion, SWIR-MPI is limited by a partial volume effect in spatially complex tissues, as probing photons may travel through different tissue types and the resulting measurements represent a weighted average of those regions. This may help explain the modest discrepancies between SWIR-MPI blood lipid measurements and invasive blood draws (Fig. 5). This effect can potentially be mitigated through the use of multi-layer inverse models or tomographic reconstructions that take different tissue regions (e.g., the skin layer) into consideration^64^. Similarly, the application of skin cream may also affect blood lipid results, and requires further investigation going forward. Additionally, the gender representation of the blood lipids study was skewed (nine males/one female). In the future, it will be important to recruit a more gender-balanced group, which will permit the investigation of questions such as how lipid metabolism is potentially different between male and female individuals. Finally, unlike optical microscopy where resolution can be diffraction limited at the sub-micron level, the spatial resolution of SWIR-MPI is limited by the diffusion length of collected photons, which is approximately sub-mm for biological tissue, depending on the optical properties^19^.

In summary, we have developed SWIR-MPI, a new label-free non-contact optical imaging modality that can spatially map the levels of water and lipids in tissue. This modality fills an important gap left by other imaging modalities through its capability to measure these chromophores in a wide-field manner without the use of exogenous agents. We have demonstrated the importance of quantitative in vivo water and lipid measurements through multiple applications that span cancer, cardiovascular disease, obesity, and others. Based on our findings, SWIR-MPI has the potential for significant impact in basic science studies and clinical patient monitoring.

## Methods

### Design of the hyperspectral SWIR-MPI system

The system diagram and data processing steps are shown in Fig. 1a. The illumination source was an ultrafast pulsed laser (InSight DS+, Spectra-Physics, Santa Clara, CA, USA), producing ~120 fs pulses at an 80 MHz repetition rate, with center wavelength tunable from 680 to 1300 nm in 1 nm increments. The average output power varied between 800 mW and 1.5 W over the spectrum. This laser was used owing to its spectral range, high output power, and because it was already available in the authors’ laboratory as a light source for a commercial multiphoton system. The pulsed laser was effectively used as a tunable continuous-wave (CW) light source. Alternatively, a CW laser, or a lamp coupled to a filter could be used as light source for the SWIR-MPI system. Meso-scale spatial patterns were generated using a DMD (CEL5500 Light Engine, Digital Light Innovations, Austin, TX, USA). A germanium-doped CMOS camera with spectral sensitivity over the 300–1600 nm range (TriWave, Infrared Laboratories, Inc., Peabody, MA, USA) was used for collection. The camera operates with −80 °C cooling, and has a pixel format of 640 × 480 with 10 µm pixel pitch. The integration time varied from milliseconds to hundreds of milliseconds across the NIR-SWIR wavelengths. A SWIR imaging lens (SR0510-A01, StingRay, Keene, NH, USA) was used with the camera. Orthogonal linear polarizers (VLR-29.2MM-NIR, VersaLight^TM^, Meadowlark Optics, Frederick, CO, USA) were used to reduce specular reflection from the tissue surface.

The laser output beam was expanded by passing through an optical phantom diffuser and a collimating lens (#65-438, Edmund Optics, Barrington, NJ, USA) before illuminating the DMD. The optical phantom diffuser effectively mitigated speckles. A 75 mm focal length projection lens (#65-439, Edmund Optics, Barrington, NJ, USA) was used to magnify and image the DMD onto the sample plane. DC (0 mm^−1^) and sinusoidal patterns (0.1 mm^−1^) were projected onto the sample with 0°, 120°, 240° phases at each illumination wavelength. Demodulated images were calculated for each of these two spatial frequencies at each wavelength using $I = \frac{{\sqrt 2 }}{3}\sqrt {\left( {\left( {I_1 - I_2} \right)^2 \,+\, \left( {I_2 - I_3} \right)^2 \,+\, \left( {I_3 - I_1} \right)^2} \right)}$^19^, where *I* is the intensity of the demodulated image and *I*-subscripts indicate the three phase shifts of the sinusoid. A genetic-algorithm was used in the demodulation to remove potential artifacts^65^. The instrument response to the different wavelengths and spatial frequencies was calculated by taking identical measurements of a sample of 10% intralipid with optical properties found in the literature^25,26^. Once calculated, this system response was removed from each sample image to obtain diffuse reflectance images. Optical absorption and reduced scattering coefficients were estimated at each illumination wavelength from a lookup table generated by Monte Carlo simulations with diffuse reflectance at 0 mm^−1^ and 0.1 mm^−1^ as inputs^19^. Concentrations of chromophores (lipid, water, oxyhemoglobin, and deoxyhemoglobin) were calculated by least squares fitting of absorption coefficient values at multiple wavelengths using known extinction coefficients for each chromophore^30^. We note that it is common practice in the diffuse optical spectroscopy field to report water and lipid concentrations as percentages (%)^20^. The water content is reported as the concentration of measured tissue water divided by pure water concentration (55.6 M). The lipid concentration is reported as relative to an assumed “pure” lipid density of 0.9 g/ml^20^. In addition, for hyperspectral measurement, the acquisition time is ~30 s per wavelength. The speed is limited by laser switching wavelengths and the Germanium camera updating dark frames. Although we find the current speed acceptable for the demonstrated applications, the speed can be significantly improved in the future by using other cameras and/or light sources, such as InGaAs camera and broadband lamp coupled to a filter.

### Water/lipid phantom fabrication and imaging

Liquid phantoms comprising specific proportions of lipid and water were made by varying ratios of water and Intralipid (2B6061, State Surgical Supply, Siloam Springs, AR). SWIR-MPI measurements were conducted on liquid phantoms ranging from 5 to 20% lipid at wavelengths from 910–1290 nm in 20 nm increments.

For the depth penetration study (Fig. 1e), the absorbing tubes and background liquid phantoms were composed of water, nigrosin (#N4754, Sigma-Aldrich, St Louis, MO), NIR dye (NIR1054WD, QCR Solutions Corp, Port St. Lucie, FL), and titanium dioxide (#248576, Sigma-Aldrich, St Louis, MO). The background phantom was made with 0.06 g/L nigrosin solution with sonicated TiO_2_. Optical property maps were measured with SWIR-MPI at 680 nm and 1100 nm. The absorbing tubes were made from hollow tubes filled with 0.05 g/L nigrosin and 0.06 g/L NIR dye solution resulting in an absorption coefficient roughly 10× that of the background at 680 nm and 1100 nm (Supplementary Fig. 3b).

### Evaluation of SWIR-MPI imaging depth

The SWIR wavelength band has reduced optical scattering in tissue compared to the VIS and NIR bands. In some cases, this can lead to improvement of imaging depth. To quantify this effect we first calculated the effective photon penetration depth during planar illumination, which is dependent on both optical absorption and scattering of tissue: $\delta _{eff}(\lambda ) = 1/\sqrt {3\mu _{\mathrm{a}}\left( \lambda \right)[\mu _{\mathrm{a}}\left( \lambda \right) + \mu _s^\prime \left( \lambda \right)]}$^19^. We identified a SWIR penetration window at wavelengths around 1100 nm based on previously reported mouse tissue optical properties (Supplementary Fig. 3c)^66,67^. The improved penetration depth at 1100 nm was confirmed with Monte Carlo simulations that took into account the spatial frequency of illuminating light (Supplementary Fig. 3d)^68^. In addition, a phantom study was conducted to demonstrate experimentally the improved penetration depth in the SWIR region compared to NIR. Glass capillary tubes containing absorbing dyes were placed at depths from 1 to 4 mm within a homogenous nigrosin-TiO_2_ phantom (Fig. 1e). The phantom was carefully fabricated to have nearly identical absorption at both 680 nm and 1100 nm so that the effect of optical scattering on imaging depth could be isolated (Supplementary Fig. 3b). The absorption maps and line profiles show that absorption contrast is apparent with SWIR-MPI at increased depths at 1100 nm compared to at 680 nm (Fig. 1f, g). In addition, the signal-to-background ratio (SBR) was higher in the SWIR penetration window at all four tube depths (Supplementary Table 5). Most importantly, the 4 mm tube was not detectable with the NIR measurement while this tube provided a 1.2 SBR when imaged with SWIR light. These results demonstrate that the SWIR penetrating window can be used to improve tissue imaging depth over VIS or NIR light. As the illumination wavelength extends beyond 1300 nm, water absorption increased dramatically, outcompeting further reductions in scattering^69^.

### In vivo water monitoring study for simulated edema

BALB/c mice (Charles River Laboratories, Cambridge, MA) were used for the study in accordance with an institutionally approved protocol and federal guidelines. Hair was removed with clippers and hair removal lotion (Nair, Church & Dwight, NJ) before measurements. Simulated edema was induced by subcutaneous injection of either 0 mL, 0.1 mL, or 0.2 mL of 10% PBS. The injection volume was based on prior work^70^. There were four mice per group. Baseline measurements were taken before injection, and longitudinal measurements were taken every 5 min for a 2-hour duration post injection. The mice were maintained under anesthesia (2% inhaled isoflurane, 98% oxygen) throughout the experiment. SWIR-MPI measurements to extract water content were conducted at wavelengths from 970 to 1270 nm in 60 nm increments. A 0.5 cm diameter area around the injection site was used as an ROI to quantify changes in water content.

### In vivo water monitoring study during acute inflammation

Over 2 weeks after the water monitoring study, the same mice were used to test the ability of SWIR-MPI to monitor inflammation. TPA, #P1585, Sigma-Aldrich, St. Louis, MO, USA) was dissolved in acetone (#179973, Sigma-Aldrich, St. Louis, MO, USA) at a concentration of 1 μg/μL. In all, 10 μL TPA solution was injected into the flank of each mouse in the group (*n* = 4) to induce acute inflammation^71,72^. The same volume of PBS was injected into each animal in the control group (*n* = 4). Baseline measurements were taken before injection, followed by measurements every 5 min for 2 h. Blood samples were extracted from the tail vein of each mouse before injections. Mice were maintained under anesthesia throughout the experiment. SWIR-MPI measurements were conducted at wavelengths from 970 to 1270 nm in 60 nm increments to extract water content. Spatial frequencies of 0 mm^−1^ and 0.1 mm^−1^ were used for SWIR-MPI measurements. Following measurement and 5 h after the injection, blood samples were extracted again via tail vein before each mouse was sacrificed. After killing each mouse, tissue surrounding the injection site was excised and fixed in formalin for histologic examination. WBC counts were conducted with a Bright-Line Hemacytometer (#1492, Hausser Scientific, Horsham, PA). Formalin-fixed tissues were sectioned at four microns and stained (H&E) by the Specialized Histopathology Core of Dana-Farber/Harvard Cancer Center. The presence of inflammation was evaluated by a board-certified pathologist (JCA) who was blinded to the source of each tissue section.

### Ex vivo mapping of tumor lipid content

The PC3/2G7 prostate tumor xenograft model was used for this study^27^. PC3/2G7 cells were grown and expanded at 37 °C in a humidified 5% CO_2_ atmosphere in RPMI-1640 culture medium containing 7% fetal bovine serum, 100 units/mL penicillin and 100 μg/mL streptomycin. Severe combined immunodeficient (SCID) hairless outbred mice (SHO MouseCrl:SHO-PrkdcscidHrhr), age 5–6 weeks old (21–23 gram), were purchased from Charles River Laboratories (Cambridge, MA), and housed in the Boston University Laboratory Animal Care Facility in accordance with an institutionally approved protocol and federal guidelines. Autoclaved cages containing food and water were changed once a week. On the day of tumor cell inoculation, 4 × 10^6^ PC3/2G7 cells were subcutaneously injected on the posterior flank of a SCID mouse in 0.2 ml serum-free RPMI using a U-100 insulin syringe with a 28.5-gauge needle. Tumor length (*L*) and width (*W*) were measured every 3 days, and the volume calculated as *V* = (*π*/6) × (*L* × *W*)^3/2^. Once the volume exceeded 3000 mm^3^, the animal was killed, and the tumor was resected and cut in half. The cut face of the tumor was imaged by SWIR-MPI at wavelengths from 910 nm to 1290 nm in 20 nm increments.

The edges of the tumor cross-sections were marked with green and yellow permanent inks to guide co-registration of microscopic sections. After imaging, the tissue was snap frozen, sectioned, and stained using Oil Red O with hematoxylin counterstain to evaluate lipid content^47^ using a bright-field microscope with ×10 objective (Nikon TE200). Cross-sections from three tumor samples were measured and stained for lipid content in this study. In each histology slide, the green ink mark was identified and a large rectangular ROI was imaged under the microscope across the tumor sample starting at that position. The yellow ink mark was also identified and was later used to co-register the stained slide with the SWIR-MPI lipid map. The use of two color ink marks was crucial to co-register the spatial orientation of the stained slide with that of the original tumor sample. In addition, during microscopic imaging, the choice of the rectangular ROI was blinded from the SWIR-MPI lipid maps. The width of the ROI was ~1.2 mm. For each tumor sample, the rectangular ROI was equally divided into 10 sub-areas to calculate lipid fractions, which were then compared with corresponding areas on the lipid content map measured by SWIR-MPI. The staining of the tissue slides was conducted by the Specialized Histopathology Core of Dana-Farber/Harvard Cancer Center.

In order to automatically identify pixels representing lipids in the microscopic images, over 1800 pixels were first manually labeled as lipid or non-lipid by visual inspection of those images. Then we compared two classification methods: simple thresholding and nearest-neighbor. The labeled data was randomly separated into training and test sets, where the training set was composed of 500 lipid pixels and 1000 non-lipid pixels, and the test set was composed of 150 lipid pixels and 150 non-lipid pixels. For the thresholding method, we identified that thresholds in the Hue channel could best separate the two classes with 93% and 91% accuracies for lipid and non-lipid, respectively on the training set. On the test set, the thresholding method had 80 and 93% performance for lipid and non-lipid samples, respectively. For the nearest-neighbor method, we first converted each pixel from RGB to HSV, and then combined both spaces into a six-channel vector with RGB channels normalized to [0, 1]. The classification was conducted by searching for the closest pixel in the training set by Euclidean distance. It achieved 100% accuracy in the training set and 98% accuracy for both lipid and non-lipid pixels in the test set. Based on the comparison between thresholding and nearest-neighbor methods, for all the microscopic images, we applied the nearest-neighbor classification on each pixel and generated binarized lipid maps where lipid pixels were labeled as 1. The lipid fractions were then calculated from those binarized maps as the ratio of the number of lipid pixels to the total number of pixels in the image. Results were compared with the % lipid content determined by SWIR-MPI imaging.

### In vivo identification of brown adipose tissue

A total of ten healthy C57BL/6 mice (Charles River Laboratories, Cambridge, MA) were included for the brown adipose tissue classification study. Adipose tissue was excised at anatomical locations known to contain BAT and WAT fat^42^. These BAT and WAT samples were imaged with SWIR-MPI at wavelengths from 900 nm to 1300 nm in 5 nm spectral increments. Under anesthesia, in vivo measurements were also conducted with the same wavelengths for the training set (*n* = 7). Optical absorption and reduced scattering from the measured wavelengths were extracted, and three wavelengths (1145 nm, 1205 nm, and 1245 nm) were identified for BAT classification. The three wavelengths were determined with the training data by using a published feature selection algorithm^43^, where a hidden variable is assigned to each wavelength and estimated from 0 to 1. The closer to 1, the more informative the feature (i.e., wavelength) is to the classification. The top three wavelengths were the ones that had the highest estimated hidden variable values. SWIR-MPI measurements on the excised tissues at these three wavelengths were then used to train a SVM classifier to identify BAT. Only data from image pixels corresponding to excised tissue was used in wavelength selection and classifier training. In addition, to prevent over-fitting, ex vivo tissue data from four mice were used for the wavelength selection and classifier training, and in vivo data from the other three mice were used for cross-validation. The SVM was then tested on an independent test set of animals (*n* = 3). Under anesthesia, in vivo measurements were conducted in the test set with the three identified wavelengths, and the same SVM was used on high-fat regions to distinguish BAT from other tissues through the intact skin^43^. The high-fat regions were determined as areas with top 30% lipid content in the generated lipid maps. A pixel-by-pixel classification was conducted within each area/patch with the trained SVM, and the high-fat patches were identified as BAT or WAT by majority-vote (flowchart of classification in Supplementary Fig. 6). Subcutaneous tissues from anatomical areas expected to contain BAT and WAT were excised, preserved, sectioned, and H&E stained for validation of the SVM classification^42^. Tissue processing and slide preparation and staining was performed by the Specialized Histopathology Core of Dana-Farber/Harvard Cancer Center.

### Pixel independence during SVM training

Training the SVM for BAT and WAT was conducted with all available pixels on the tissue measured by SWIR-MPI. However, individual pixels are likely to exhibit spatial correlations with adjacent pixels and therefore cannot be considered statistically independent, as a result of the diffuse nature of light propagation in tissue. The extent of the spatial correlation can be assessed by taking the inverse Fourier transform of the modulation transfer function in the spatial frequency domain (i.e., diffuse reflectance as a function of spatial frequency), which gives us the point spread function (PSF) of the medium in the spatial domain^19^. The PSF was calculated with average *μ*_a_ and *μ*_s_′ values of 0.05 mm^−1^ and 1.0 mm^−1^, respectively, estimated from all wavelengths over the course of the brown fat experiment in the training phase, and arrived at a full-width half-maximum (FWHM) value of ~0.6 mm for the spatial PSF. Alternatively, the spatial dependence can be assessed through the estimation of the transport mean free path $\left( {l_t^\prime = \frac{1}{{\mu _{\mathrm{a}} + \mu _s^\prime }}} \right)$, the mean distance after which a photon’s direction becomes random^24,73^. The $l_t^\prime$ computed for this study was ~1 mm, which is typical in biological tissues^73^. Based on these estimates, we considered pixels ≥1.2 mm apart to be “optically independent pixels”. This distance is larger than the $l_t^\prime$, and twice the FWHM of the spatial PSF. We repeated the SVM training for the brown fat classification by downsampling the SWIR-MPI images with a 1.2 mm × 1.2 mm grid, which led to ~3600 (optically independent) pixels from the original 177,000 pixels. We found both classifiers were able to successfully identify the interscapular brown fat regions in the test set.

### Non-invasive blood lipid monitoring in human subjects

This study was conducted in accordance with an institutionally approved protocol and federal guidelines. Informed consent was obtained from all participants prior to the experiment. Ten human subjects were recruited (nine males and one female, with an age distribution of 36 ± 15). The human subjects fasted for 10-hours overnight then were provided a high-fat meal. The high-fat meal was either one Uno’s personal pizza or a McDonald’s breakfast sandwich, both containing approximately the same amount of total fat content (60 g). Subjects were free to choose which meal to eat. A total of ten subjects were measured with SWIR-MPI in the morning (before the high-fat meal) and afternoon (5 h after the meal) using wavelengths from 730 nm to 1250 nm in 40 nm increments. The measurements were conducted on the dorsal surface of the hand. Blood draws were conducted immediately after each SWIR-MPI measurement by a trained phlebotomist (Boston Clinical Laboratories, Inc., MA) with lipid panel analysis performed by the same company. The total blood lipid content from the lipid panel (triglycerides+total cholesterol) was correlated with the average SWIR-MPI index. The average SWIR-MPI index value was calculated by averaging over pixels corresponding to superficial veins on the subject’s hand. The superficial veins were identified by pixels corresponding to the top 10% total hemoglobin content in the segmented vessel areas. The vessels were automatically identified over the whole hand region by applying Hessian-based multiscale filtering (implemented with the MATLAB built-in “fibermetric” command) on total hemoglobin concentration maps extracted with NIR wavelengths. The average percentage error was calculated by comparing the percentage difference between the lipid value given by the linear fit and the blood draw results. In order to generate the Δ SWIR-MPI index map, the 810 nm planar illumination images were manually co-registered at the two time points to account for the relative spatial translation and rotation of the subject’s hand. The Δ SWIR-MPI index map was calculated by subtracting the index map obtained in the afternoon measurement from that of the morning measurement. With respect to relative motion between two measurements, only the commonly imaged hand area is visualized for delta SWIR-MPI index.

### Statistical tests

A one-sample *t* test was used in the water monitoring study to compare water content of before versus after the injection. A two-sample *t* test was used in the inflammation study to compare between the PBS and TPA groups.

### Reporting summary

Further information on research design is available in the Nature Research Reporting Summary linked to this article.

## Supplementary information

Supplementary Information

Reporting Summary

## Data Availability

All the relevant data supporting the findings of this study are available within the paper, its Supplementary Information, and from the corresponding author upon reasonable request. Source data are provided with this paper.

## Source data

Source Data

## Code availability

All the relevant scripts supporting this study are available from the corresponding author upon reasonable request, and are publicly available for download in the Github repository: https://github.com/zhaoyy89/SWIR-MPI.
