# Supplementary material for: Shortwave-infrared meso-patterned imaging enables label-free mapping of tissue water and lipid content
Source: Nat Commun. 2020 Oct 23;11:5355. doi: 10.1038/s41467-020-19128-7 (PMC7585425; doi:10.1038/s41467-020-19128-7)
Supplement: Supplementary file 1 — Supplementary Information [file 41467_2020_19128_MOESM1_ESM.pdf]

Shortwave-infrared meso-patterned imaging enables label-free mapping of tissue water and lipid content

Authors:
Yanyu Zhao[1,2], Anahita Pilvar[1], Anup Tank[1], Hannah Peterson[1], John Jiang[1], Jon C. Aster[3], John Paul Dumas[4], Mark C. Pierce[4], Darren Roblyer[1,*]

[1] Department of Biomedical Engineering, Boston University, 44 Cummington Mall, Boston, Massachusetts 02215, USA
[2] Beijing Advanced Innovation Center for Biomedical Engineering, School of Biological Science and Medical Engineering, Beihang University, Beijing, 100191, China
[3] Department of Pathology, Brigham and Women's Hospital and Harvard Medical School, 75 Francis Street, Boston, MA 02115
[4] Department of Biomedical Engineering, Rutgers, The State University of New Jersey, 599 Taylor Road, Piscataway, NJ 08854, USA
** roblyer@bu.edu*

Supplementary Note 1 – Comparison of SWIR-MPI with other diffuse optical technologies

Here SWIR-MPI is explicitly compared to other diffuse optical technologies including Spatial Frequency Domain Imaging (i.e. SFDI) and Diffuse Optical Spectroscopic Imaging (i.e. DOSI)[1–4].

1. Table and figure comparing DOSI, SFDI, and SWIR-MPI

Table S1 compares SFDI, DOSI and SWIR-MPI over several quantitative parameters (wavelength, spatial resolution, measurement type, chromophores available). Supplementary Figure 1 shows the normalized absorption coefficient of oxy-hemoglobin, deoxy-hemoglobin, water, and lipids at different wavelengths. The shaded areas indicate the wavelength regions utilized by SFDI, DOSI, and SWIR-MPI, respectively. SFDI works use 640 – 851 nm wavelengths in the NIR range (known as the first *in vivo* imaging window), which don't have access to water and lipid absorption peaks/features[1,5,6]. On the other hand, DOSI uses 650 – 1,000 nm wavelength region which is mostly in the NIR, and it also uses the first *in vivo* imaging window[2–4,6]. In contrast, SWIR-MPI utilizes 680 – 1,300 nm wavelengths that cover large range of both NIR and SWIR, and can access the well-known second *in vivo* imaging window[6]. In terms of chromophores, both DOSI and SWIR-MPI can extract oxy-hemoglobin, deoxy-hemoglobin, water and lipids, whereas SFDI is only able to extract oxy- and deoxy-hemoglobins. For spatial resolution, compared to DOSI, both SFDI and SWIR-MPI uses structured illumination in the spatial frequency domain which has sub-millimeter spatial resolution compared to DOSI whose resolution is reported to be approximately 0.5 – 1 cm[1,4]. In addition, the measurement of SWIR-MPI is conducted in a wide-field, non-contact manner, which doesn't mechanically interfere with the tissue. In contrast, DOSI measurement is generally conducted with a 1 cm × 1 cm grid, and the measurement requires mechanical contact between the tissue and optical probe.

2. Water and lipid extraction simulations – wavelength comparison

To further compare water and lipid content extraction with different wavelengths utilized by SFDI, DOSI, and SWIR-MPI, representative chromophore concentrations in a physiological range were identified based on previous literature[2–4,7]. Specifically, we consider water content in the range of 16.6 % - 100 %, lipid content in the range of 40 % - 80 %, oxy-hemoglobin concentration in the range of 13.3 – 150 μM, and deoxy-hemoglobin concentration in the range of 4 – 50 μM[2–4,7]. 10,000 sets of chromophore concentrations (oxy-hemoglobin, deoxy- hemoglobin, water, and lipids) were then randomly generated from the above ranges. Then, corresponding absorption spectra were generated, and zero mean Gaussian noise was added to each absorption value. Finally, chromophore concentrations were calculated and compared to the ground truth in terms of percent errors. The mean and standard deviation of percent error for water and lipids are summarized in Table S2, with different wavelength combination and absorption spectra noise levels. The wavelength comparison shows that the SWIR wavelengths utilized by SWIR-MPI achieve higher accuracy for water and lipids extractions compared to the other wavelength combinations used by SFDI and DOSI. For example, under 1% Gaussian noise, DOSI with 351 wavelengths in the range of 650 – 1,000 nm has percent errors of 0.0 ±0.6 % for water and 0.0 ±2.1 % for lipids, respectively. In contrast, with 81 wavelengths in the range of 900 – 1,300 nm, SWIR-MPI has percent errors of 0.0 ±0.4 % for water and 0.0 ±0.9 % for lipids, respectively. SWIR-MPI also achieves the lowest

errors under 5% and 10% Gaussian noise levels, with an especially large advantage in lipid extractions errors.

3. Example of spatial resolution comparison between DOSI and SWIR-MPI

While both DOSI and SWIR-MPI have access to water and lipids, measurements of the two modalities with the same sample were compared side-by-side, which demonstrates that SWIR-MPI is able to reveal significantly more spatial details. Specifically, a pork tissue with relatively rich spatial features (i.e. fat deposits) was measured using both modalities. Supplementary figure 2a shows white light image of the pork tissue. The rectangular dashed boxes indicate regions where DOSI and SWIR-MPI measurements were conducted. Fig. S2b,c are water and lipid maps as well as optical property maps (930 nm) from DOSI with two different measurement settings. The optical property maps were generated using linear interpolation of the measured individual points as in prior literature[2–4]. The DOSI measurements were conducted following a rectangular grid pattern with a fixed spacing between measurement points. In addition, the measurements were taken with 658, 690, 785, 808, 830, and 850nm in the 50 – 400 MHz frequency range, 650 – 1,000 nm continuous-wave, and 28 mm or 10 mm source-detector separation. For Fig. S2b, we used measurement settings of 10 mm grid spacing and 28 mm source-detector separation, which was in consistency with DOSI literature[2–4]. To provide additional comparisons, for Fig. S2c we further reduced the source-detector separation to 10 mm and conducted measurements with a 5×10mm grid spacing. The dark blue areas in DOSI maps were related to removal of some measurements due to non-physiological values arising in data processing. (For example if the calculated scattering parameter "b" resulted in a positive slope for the wavelength dependence of scattering). With the measured points, DOSI was able to provide some spatial contrast of the sample. Supplementary Figure 2d shows water and lipid maps as well as optical property maps (1,210 nm) from SWIR-MPI, where substantially more spatial details are revealed. The extracted water and lipid values by the two modalities shows moderate discrepancy due to the partial volume effect on the spatially complex sample and different probing depths. The side-by-side comparison shows that SWIR-MPI is able to provide much finer spatial information due to the superior spatial resolution compared to DOSI.

Overall, these combined analyses show that SWIR-MPI has higher spatial resolution than DOSI, and a better ability to accurately extract water and lipids compared to SFDI and DOSI, even when many more wavelengths are used in the SFDI and DOSI methodologies.

Supplementary Note 2 – System Calibration

SWIR-MPI system calibration was conducted by imaging the sample and the calibration phantom under identical illumination and acquisition conditions at each wavelength of interest. The calibration phantom was 10% intralipid which has well-established optical properties[8,9]. At each illumination wavelength, DC (0 mm^{-1}) and sinusoidal patterns (0.1 mm^{-1}) displayed on the DMD were projected with phases of 0°, 120°, and 240°. Demodulated images were calculated for each of these two spatial frequencies at each illumination wavelength using $I = \frac{\sqrt{2}}{3}\sqrt{((I_1 - I_2)^2 + (I_2 - I_3)^2 + (I_3 - I_1)^2)}$ [1], where I is the intensity of the demodulated image and I_1, I_2, and I_3 represent raw images acquired for each of the phase shifted illumination patterns.

At each wavelength, the demodulated images of the sample and calibration phantom at spatial frequency f_x (i.e., $I_{sample}(f_x)$ and $I_{calibration}(f_x)$, respectively) were used to calculate the diffuse reflectance of the sample at each pixel, denoted as $R_{d\_sample}(f_x)$, (Eq. 1). This process also corrects for any non-uniformity in the illumination intensity or sensitivity over the CMOS pixel array. The diffuse reflectance of the calibration phantom, denoted as $R_{d\_calibration}(f_x)$, was pre-computed by Monte Carlo simulations (anisotropy g of 0.7 and refractive index n of 1.33) which map the known phantom optical properties to diffuse reflectance values at each spatial frequency used for illumination[10,11].

$$R_{d\_sample}(f_x) = \frac{I_{sample}(f_x)}{I_{calibration}(f_x)} * R_{d\_calibration}(f_x) \quad (1)$$

These diffuse reflectance measurements were then used as input to an inverse model that calculates tissue optical properties on a pixel-by-pixel basis. The inversion algorithm searches a pre-computed lookup table (LUT) generated by Monte Carlo simulations of light propagation in tissue (anisotropy g of 0.9 and refractive index n of 1.43)[10]. The LUT ultimately maps the diffuse reflectance values measured at each spatial frequency to unique combinations of optical absorption and reduced scattering (i.e., μ_a and μ_s').

Supplementary Note 3 – Drift test for measurement robustness

In order to test the robustness, a drift test was conducted over a course of 5 hours. Specifically, 10% lipid phantom was repeatedly measured over 5 hours with 1-hour time interval, and the water and lipid concentrations were extracted. The calibration measurement was conducted at the beginning of the drift test, followed by the phantom measurements in the next multiple hours. The measurements were conducted using 910 – 1,290 nm wavelengths with 20 nm increments as well as 0 and 0.1 mm^{-1} spatial frequencies. The extracted optical properties are compared with expected values in the Supplementary Figure 4 (a) and (b). The mean and standard deviation of the measured optical absorption (μ_a) and reduced scattering (μ_s') are plotted on top of the expected optical property spectra, which shows good agreement with ground truth values and small variation between measurements (i.e. relatively small standard deviations). The coefficient of variation (%), defined as the ratio of the standard deviation to the mean, is calculated for the optical absorption and reduced scattering in Supplementary Figure 4 (c) and (d), respectively. The results show coefficient of variation of 1.6±0.9% for optical absorption measurements and 1.2±0.4% for reduced scattering measurements. In addition, the extracted water and lipid concentrations of the drift measurements are compared with the known concentrations in Table S4, which also demonstrates good agreement with the ground truth, with an average error of 0.8±0.9% for water and 0.6±0.9% for lipid.

Supplementary Note 4 – Monte Carlo Simulations

The improved penetration depths achievable in the SWIR window were verified by Monte Carlo simulations, following the work of Hayakawa et al. (2018)[12]. Specifically, the Monte Carlo simulations were conducted directly in the spatial frequency domain for a semi-infinite

homogeneous diffusive medium at two competing wavelengths (i.e. 680 nm and 1,100 nm respectively)[12]. N = 1×10^8 photon packets were launched and photon visitation depths were recorded. The optical properties of the background liquid phantom in the phantom imaging depth experiment (Figure 1e) were used for the simulations. The absorption coefficient (μ_a) was 0.04 mm^{-1} at both 680 nm and 1,100 nm. The reduced scattering coefficients (μ_s') were 4.5 mm^{-1} and 1.7 mm^{-1} at 680 nm and 1,100 nm, respectively. An anisotropy of g = 0.8 and refractive index of n = 1.4 were used in all simulations. The 90th percentiles of "maximum photon visitation depths" were reported from the Monte Carlo simulations and are presented in Supplementary Figure 3d. The depth statistics indicate a 1.3 mm probing depth for planar illumination at 680 nm, compared to a 2.4 mm probing depth at 1,100 nm.

SUPPLEMENTARY FIGURES

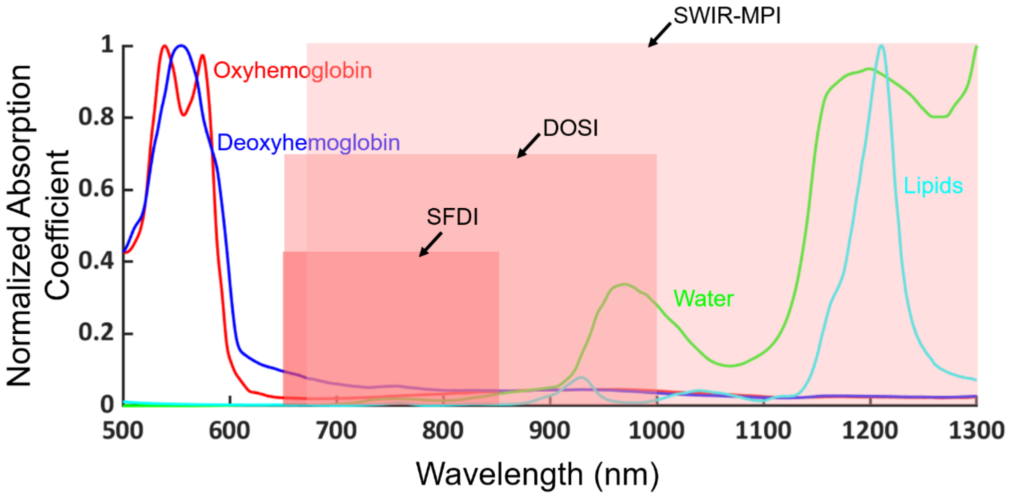

Supplementary Figure 1. Normalized absorption coefficients of oxy-hemoglobin, deoxy-hemoglobin, water, and lipids at different wavelengths[6,8,9]. The shaded areas indicate the wavelength regions utilized by different diffuse optical techniques including SFDI, DOSI, and SWIR-MPI, respectively[1–5]. Compared to DOSI, SWIR-MPI covers the water and lipid absorption peaks in the 900 – 1,000 nm range as well as in the longer SWIR wavelengths up to 1,300 nm.

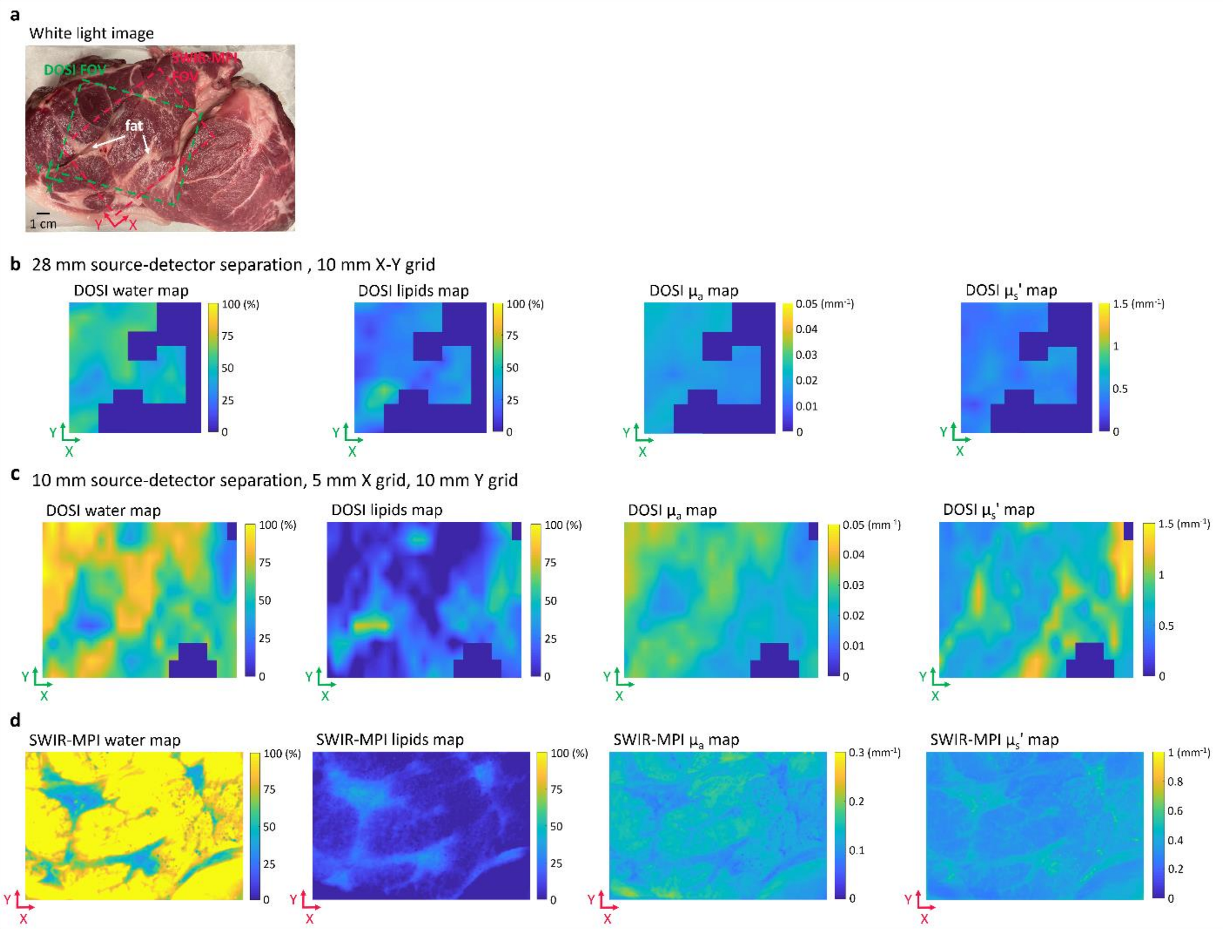

Supplementary Figure 2. a. White light image of pork belly tissue measured by DOSI and SWIR-MPI. The measurement field-of-view of DOSI and SWIR-MPI are indicated by the green and red outlined regions, respectively. **b.** Water and lipid maps, as well as optical property maps (930 nm) measured by DOSI using 10 mm grid and 28 mm source-detector separation. **c.** Water and lipid maps, as well optical property maps (930 nm) measured by DOSI using 5×10 mm grid and 10 mm source-detector separation for additional comparison. **d.** Water and lipid maps, as well optical property maps (1,210 nm) measured by SWIR-MPI.

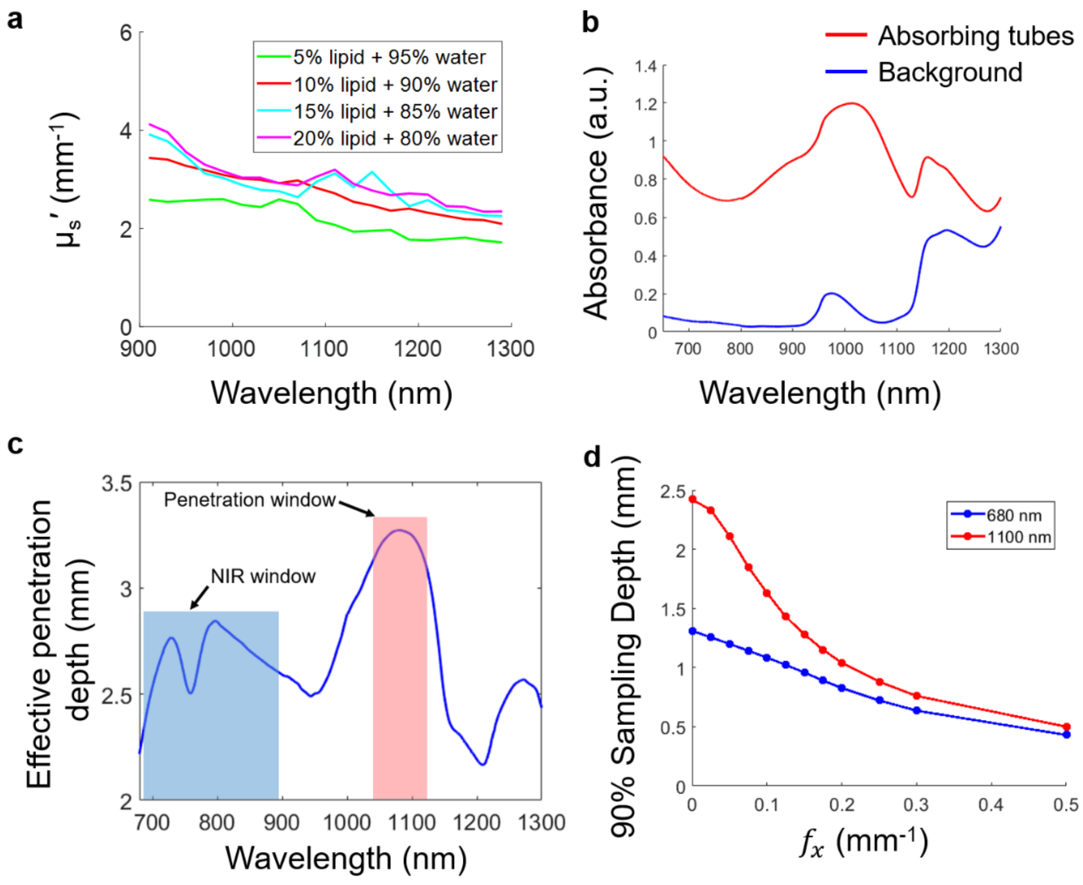

Supplementary Figure 3. a. Optical scattering (μ_s') spectra of water-lipid phantoms over a range of concentrations.. **b.** The measured absorbance spectra of the solution in the absorbing tubes (red line) and background liquid phantom (blue line), acquired with spectrophotometer measurements. The solution in the absorbing tubes was composed of nigrosin, water, and NIR dye. **c.** A SWIR penetration window near 1,100 nm was identified by calculating effective penetration depth using optical properties previously measured in mice[13]. The SWIR band centered at approximately 1,100 nm shows improved penetration depth compared to the NIR window. **d.** The improved penetration depths in the SWIR window were verified by Monte Carlo simulations conducted in the spatial frequency domain. Depth statistics for both wavelengths and spatial frequencies are presented as 90% photon visitation depths[12]. The depth statistics show 1.3 mm probing depth at planar illumination for 680 nm and 2.4 mm depth for 1,100 nm, respectively.

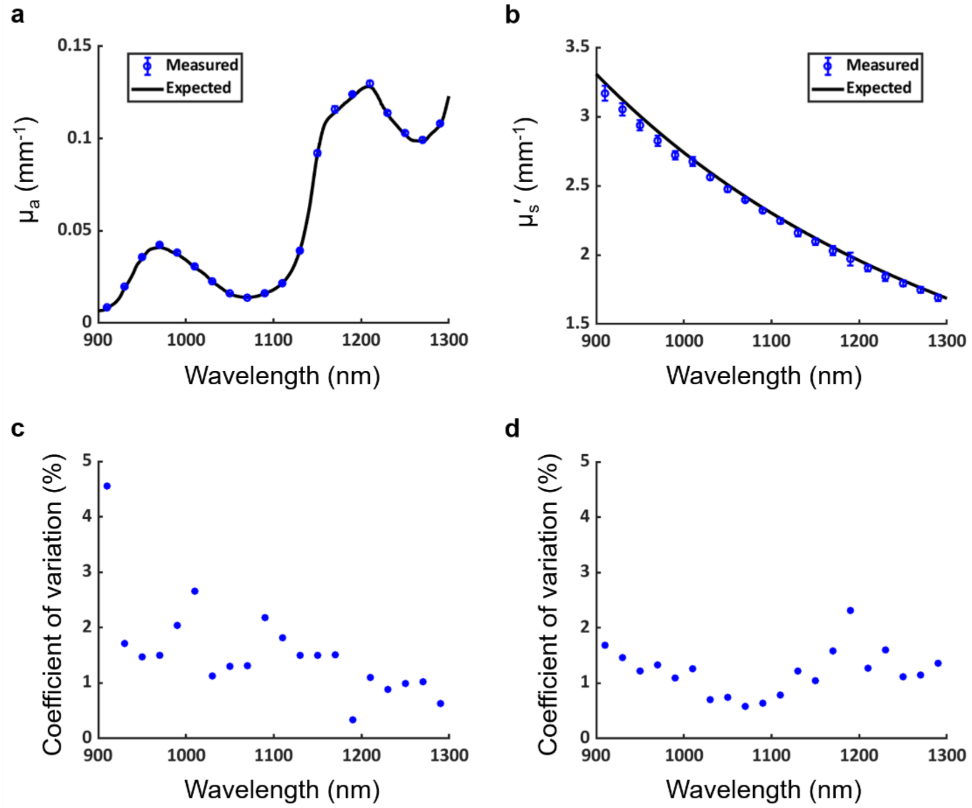

Supplementary Figure 4. a. Error bar plot of the measured and expected optical absorption during the drift test. **b.** Error bar plot of the measured and expected optical reduced scattering during the drift test. **c.** Coefficient of variation (%) of optical absorption measurements during the drift test, with an average of 1.6±0.9%. **d.** Coefficient of variation (%) of optical reduced scattering measurements during the drift test, with an average of 1.2±0.4%. Source data are provided as a Source Data file.

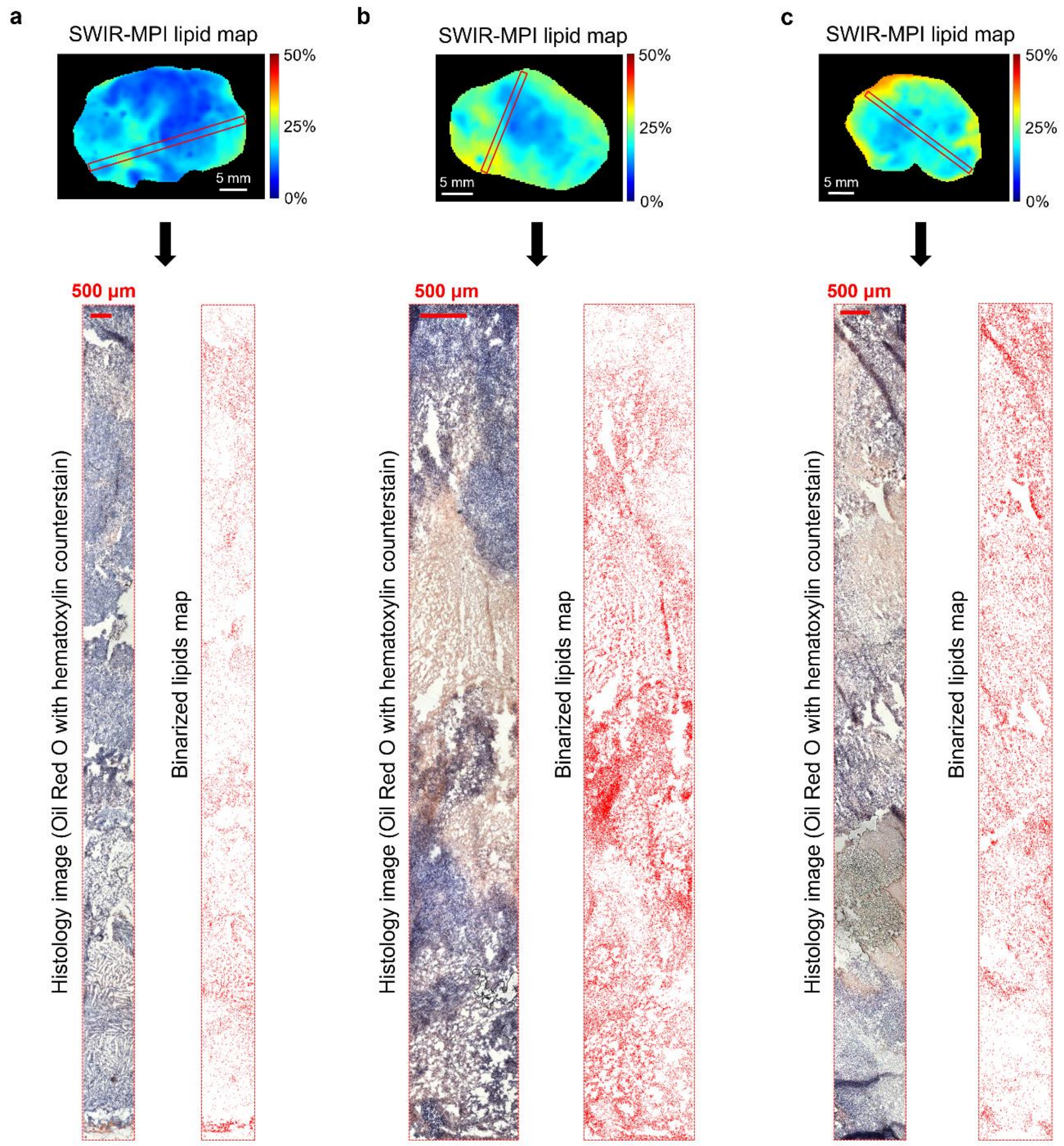

Supplementary Figure 5. Tumour cross-section lipid maps from SWRI-MPI and histology microscopic images.

a. SWIR-MPI lipid map of tumour #1 in Fig. 3e. Histology image and corresponding binarized lipid map are shown for the red rectangular ROI. The large rectangular ROI was equally divided into 10 sub-regions to calculate lipids fraction and correlated with corresponding areas on the SWIR-MPI lipid map. **b.** SWIR-MPI lipid map of tumour #2 in Fig. 3e. **c.** SWIR-MPI lipid map of tumour #3 in Fig. 3e.

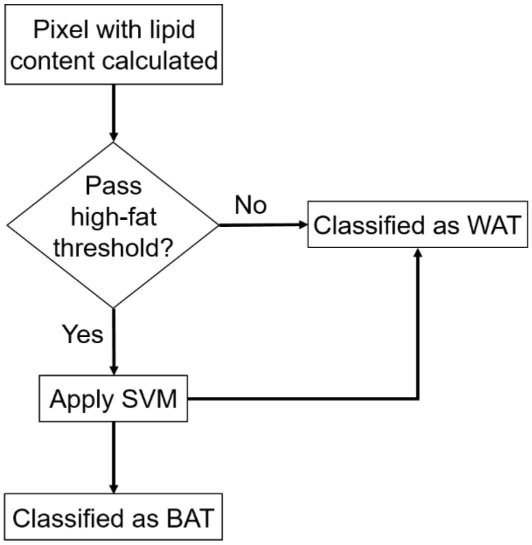

Supplementary Figure 6. Flowchart of brown and white fat classification. For each pixel in the mouse fur-free area, we first test whether its corresponding lipid concentration passes the high-fat threshold (determined in the training step as top 30% lipid content). If not, the pixel is classified as white fat (WAT). If it passes the high-fat threshold, we further apply the trained SVM, and classify the pixel into either brown fat (BAT) or white fat (WAT).

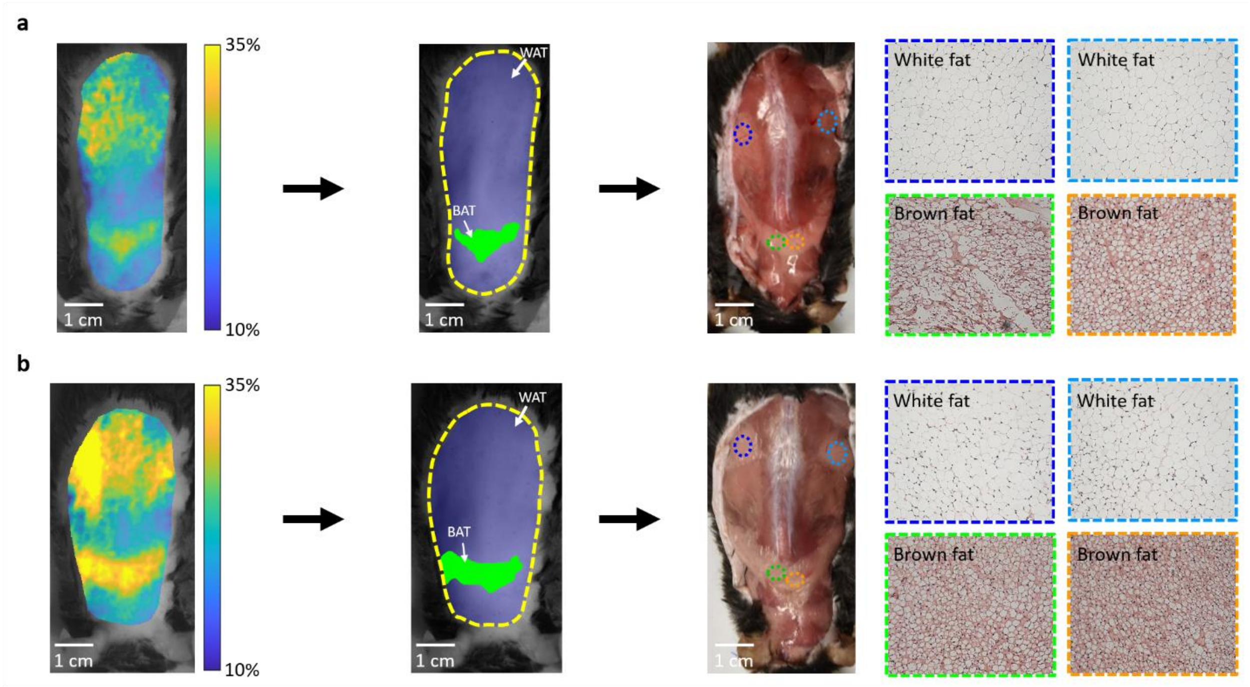

Supplementary Figure 7. Classification and verification of *in vivo* brown fat identification for the remaining two mice in the test set.

SUPPLEMENTARY TABLES

Supplementary Table 1. Comparisons between SFDI, DOSI, and SWIR-MPI.

Modality	Wavelength	Spectral range	Imaging windows	Spatial resolution	Measurement	Chromophores
SFDI	640 – 851 nm [1,5]	NIR [14–19]	First *in vivo* imaging window [6]	< 1 mm [1]	Wide-field, non-contact	Oxy- and deoxy-hemoglobin [1,5]
DOSI	650 – 1,000 nm [2–4]	NIR [14–19]	First *in vivo* imaging window [6]	0.5 – 1 cm [4]	Point-by-point, contact	Oxy- and deoxy-hemoglobin, water, lipid [2–4]
SWIR-MPI	680 – 1,300 nm	NIR + SWIR [14–19]	Second *in vivo* imaging window [6]	< 1 mm [1]	Wide-field, non-contact	Oxy- and deoxy-hemoglobin, water, lipid

Supplementary Table 2. Comparison of water and lipids extraction errors between different modalities and wavelengths.

Modalities	Wavelength	Citation	1 % Gaussian noise		5 % Gaussian noise		10 % Gaussian noise	
			Water	Lipids	Water	Lipids	Water	Lipids
SFDI (4 wavelengths)	659, 691, 731, and 851 nm	Tabassum et al.[20]	-4.9 ± 510.9 %	10.8 ± 1,327.4 %	-33.3 ± 2,517.0 %	30.0 ± 6,634.4 %	59.0 ± 5,043.9 %	-229.9 ± 13,419.7 %
SFDI (212 wavelengths)	640 – 1 – 851 nm	theoretical	0.3 ± 14.4 %	-0.7 ± 25.8 %	0.4 ± 71.3 %	-0.1 ± 129.3 %	1.2 ± 140.9 %	-1.9 ± 256.0 %
DOSI (FDPM, 6 wavelengths)	660, 680, 780, 810, 830, and 850 nm	O'Sullivan et al.[3]	0.9 ± 80.6 %	-0.7 ± 300.1 %	-4.5 ± 405.8 %	0.4 ± 1,494.6 %	15.7 ± 818.8 %	4.4 ± 2,976.6 %
DOSI (FDPM + CW, 351 wavelengths)	650 – 1 – 1,000 nm	Yazdi et al.[2] O'Sullivan et al.[3] Cerussi et al.[4]	0.0 ± 0.6 %	0.0 ± 2.1 %	0.0 ± 2.8 %	0.0 ± 10.6 %	0.0 ± 5.6 %	0.1 ± 20.9 %
**SWIR-MPI**	**900 – 5 – 1,300 nm**	**this study**	**0.0 ± 0.4 %**	**0.0 ± 0.9 %**	**0.0 ± 2.2 %**	**0.0 ± 4.7 %**	**0.0 ± 4.5 %**	**0.0 ± 9.1 %**

Supplementary Table 3. Extracted water and lipid content from water-lipid phantoms. Water and lipid concentrations were estimated from SWIR-MPI measurements by fitting the optical absorption spectra to known extinction spectra. These measured concentrations were then compared with the recipe concentrations of the phantoms. A number of three phantoms were fabricated for each concentration. The average error for water calculated over all phantoms was -0.2±2.5%, and the average error for lipid was 0.3±1.6%.

**Known content**		**Extracted content**		**Error**	
Water	Lipid	Water	Lipid	Water	Lipid
95.0%	5.0%	97.3±2.5%	3.3±0.6%	2.3±2.5%	-1.7±0.6%
90.0%	10.0%	89.1±1.4%	10.8±0.3%	-0.9±1.4%	0.8±0.3%
85.0%	15.0%	84.1±3.4%	15.2±1.4%	-0.9±3.4%	0.2±1.4%

80.0%	20.0%	78.6 ±1.5%	22.0 ±0.9%	-1.4 ±1.5%	2.0 ±0.9%

Supplementary Table 4. Accuracies of water and lipid extraction in the drift measurements.

**Drift measurements**	**Known concentration** Water	Lipid	**Extracted concentration** Water	Lipid	**Error** Water	Lipid
#1	90.0%	10.0%	89.9%	10.2%	-0.1%	0.2%
#2	90.0%	10.0%	90.0%	11.8%	0.0%	1.8%
#3	90.0%	10.0%	90.9%	11.0%	0.9%	1.0%
#4	90.0%	10.0%	91.3%	10.4%	1.3%	0.4%
#5	90.0%	10.0%	92.0%	9.4%	2.0%	-0.6%
**Average (± standard deviation)**	90.0%	10.0%	90.8% (±0.9%)	10.6% (±0.9%)	0.8% (±0.9%)	0.6% (±0.9%)

Supplementary Table 5. Quantitative comparison of signal-to-background ratio (SBR) at 680 nm and 1,100 nm.

**SBR**	**1 mm**	**2 mm**	**3 mm**	**4 mm**
680 nm	9.0	3.7	1.1	1.0
1,100 nm	10.4	5.6	1.7	1.2
Improvement	15.6%	51.4%	54.6%	20.0%

Supplementary Table 6. White blood cell counts before and 5 hours after PBS or TPA injections.

Unit: K/μl

Mice	PBS #1	#2	#3	#4	TPA #1	#2	#3	#4
Before	13,200	13,700	14,400	9,800	12,500	11,800	9,800	12,200
After	11,000	11,200	11,200	9,700	18,000	13,300	12,500	14,500

Supplementary Table 7. Rating for early signs of acute inflammation provided by a blinded board-certified pathologist. “P” refers to positive (found signs of inflammation in tissue H&E slide), and “N” refers to negative (not found signs of inflammation in tissue H&E slide).

Group	PBS (control)				TPA			
Mouse #	1	2	3	4	1	2	3	4
Pathologist decision of the resected tissue	N	N	N	N	P	P	P	N

Supplementary Table 8. Lipid panel from blood draws for all human subjects.

Subjects	Triglycerides (mg/dL)		Total cholesterol (mg/dL)		HDL cholesterol (mg/dL)		LDL cholesterol (mg/dL)	
	Morning	Afternoon	Morning	Afternoon	Morning	Afternoon	Morning	Afternoon
#01	52	61	160	159	61	60	81	77
#02	135	290	167	169	46	42	89	88
#03	61	142	111	119	60	60	35	36
#04	136	182	227	219	46	49	126	126
#05	163	159	184	182	63	62	96	91
#06	80	253	144	146	62	55	46	44
#07	46	56	199	193	64	62	125	118
#08	92	141	170	166	62	57	89	84
#09	64	82	190	188	49	47	119	118
#10	76	105	154	156	52	54	83	82

Supplementary References

1. Cuccia, D. J., Bevilacqua, F., Durkin, A. J., Ayers, F. R. & Tromberg, B. J. Quantitation and mapping of tissue optical properties using modulated imaging. *J. Biomed. Opt.* **14**, 024012 (2009).
2. Yazdi, H. S. *et al.* Mapping breast cancer blood flow index, composition, and metabolism in a human subject using combined diffuse optical spectroscopic imaging and diffuse correlation spectroscopy. *J. Biomed. Opt.* **22**, 045003 (2017).
3. O'Sullivan, T. D. *et al.* Optical imaging correlates with magnetic resonance imaging breast density and reveals composition changes during neoadjuvant chemotherapy. *Breast Cancer Res.* **15**, 1–15 (2013).
4. Cerussi, A. E. *et al.* Diffuse optical spectroscopic imaging correlates with final pathological response in breast cancer neoadjuvant chemotherapy. *Philos. Trans. A. Math. Phys. Eng. Sci.* **369**, 4512–30 (2011).
5. Karrobi, K., Tank, A., Tabassum, S., Pera, V. & Roblyer, D. Diffuse and nonlinear imaging of multiscale vascular parameters for in vivo monitoring of preclinical mammary tumors. *J. Biophotonics* **12**, 1–13 (2019).
6. Smith, A. M., Mancini, M. C. & Nie, S. Bioimaging: Second window for in vivo imaging. *Nat. Nanotechnol.* **4**, 710–711 (2009).
7. Mazhar, A. *et al.* Wavelength optimization for rapid chromophore mapping using spatial frequency domain imaging. *J. Biomed. Opt.* **15**, 061716 (2010).
8. Beckman Laser Institute. NIR Tissue Absorption. http://dosi.bli.uci.edu/research/.
9. Allen, T. J., Hall, A., Dhillon, A. P., Owen, J. S. & Beard, P. C. Spectroscopic photoacoustic imaging of lipid-rich plaques in the human aorta in the 740 to 1400 nm wavelength range. *J. Biomed. Opt.* **17**, 061209 (2012).
10. Martinelli, M. *et al.* Analysis of single Monte Carlo methods for prediction of reflectance from turbid media. *Opt. Express* **19**, 19627 (2011).

11. Flock, S. T., Jacques, S. L., Wilson, B. C., Star, W. M. & van Gemert, M. J. C. Optical properties of intralipid: A phantom medium for light propagation studies. *Lasers Surg. Med.* **12**, 510–519 (1992).
12. Hayakawa, C. K. Optical sampling depth in the spatial frequency domain. *J. Biomed. Opt.* **23**, 1 (2018).
13. Lin, A. J. *et al.* Spatial frequency domain imaging of intrinsic optical property contrast in a mouse model of alzheimer's disease. *Ann. Biomed. Eng.* **39**, 1349–1357 (2011).
14. Liang, Y. *et al.* New function of the Yb 3 + ion as an ef fi cient emitter of persistent luminescence in the short-wave infrared. *Light Sci. Appl.* 1–6 (2016) doi:10.1038/lsa.2016.124.
15. Qi, J. *et al.* Real-Time and High-Resolution Bioimaging with Bright Aggregation-Induced Emission Dots in Short-Wave Infrared Region. *Adv. Mater.* **1706856**, 1–9 (2018).
16. Thimsen, E., Sadtler, B. & Berezin, M. Y. Shortwave-infrared (SWIR) emitters for biological imaging: A review of challenges and opportunities. *Nanophotonics* **6**, 1043–1054 (2017).
17. Paluchowski, L. A., Misimi, E., Grimsmo, L. & Randeberg, L. L. Towards automated sorting of Atlantic cod (Gadus morhua) roe , milt , and liver – Spectral characterization and classification using visible and near-infrared hyperspectral imaging. *Food Control* **62**, 337–345 (2016).
18. Randeberg, L. L. Hyperspectral characterization of tissue in the SWIR spectral range: a road to new insight? 32 (2019) doi:10.1117/12.2504297.
19. Randeberg, L. L. & Hernandez-Palacios, J. Hyperspectral imaging of bruises in the SWIR spectral region. 82070N (2012) doi:10.1117/12.909137.
20. Tabassum, S. *et al.* Feasibility of spatial frequency domain imaging (SFDI) for optically characterizing a preclinical oncology model. *Biomed. Opt. Express* **7**, 4154 (2016).
